# Cavity and waveguide quantum electrodynamics with atoms and optical nanofibers

**DOI:** 10.1186/s40580-026-00565-x

**Published:** 2026-07-28

**Authors:** Karen E. Webb, Samuel K. Ruddell, Tim Keller, Jameesh Keloth, Ratnesh K. Gupta, Mitsuyoshi Takahata, Takao Aoki

**Affiliations:** 1 Department of Applied Physics, Waseda University, 3-4-1 Okubo, Shinjuku, Tokyo, 169-8555 Japan; 2 RIKEN Center for Quantum Computing (RQC), RIKEN, 2-1 Hirosawa, Wako, Saitama 351-0198 Japan

## Abstract

Optical nanofibers provide a versatile platform for exploring strong light–matter interactions at the single-quantum level, a central goal of quantum optics. Their subwavelength diameter supports tightly confined guided modes with strong evanescent fields, enabling efficient coupling between photons and nearby atoms. By incorporating fiber Bragg gratings, nanofibers can form fully fiber-integrated high-finesse cavities, combining strong atom–photon coupling with efficient and low-loss optical input–output interfacing in the setting of cavity quantum electrodynamics (QED). In parallel, nanofiber systems naturally realize waveguide QED, where atoms interact strongly with propagating photons in a one-dimensional geometry. In this review, we introduce the underlying physical principles, summarize key experimental techniques and representative phenomena, and discuss prospects for applications in quantum information processing, quantum networks, and related quantum technologies.

**Graphical abstract:**

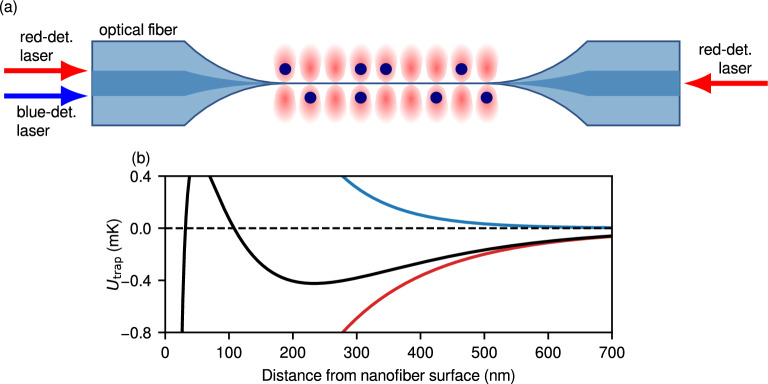

## Introduction

Quantum electrodynamics (QED) provides the most fundamental theoretical framework for describing the interaction between light and matter, and its experimental exploration has long been a central theme in quantum optics. A major breakthrough in this field was achieved by introducing optical resonators that confine photons within a spatially small volume, thereby enhancing the interaction between a single atom and a single photon. This development gave rise to the field of cavity quantum electrodynamics (cavity QED). In the strong-coupling regime of cavity QED, the presence of a single photon can dramatically alter the response of an atom, while conversely a single atom can strongly modify the transmission and reflection properties of the cavity. Such regimes enable direct control of coherent light–matter interactions at the level of individual quanta, opening pathways toward applications in quantum information processing, quantum networks, and quantum-enhanced metrology.

Traditionally, experimental cavity QED has been realized using free-space Fabry–Pérot cavities or dielectric whispering-gallery-mode cavities such as microspheres and microtoroids. These platforms have successfully achieved high quality factors and small mode volumes, satisfying the conditions for strong coupling. At the same time, each of these systems faces practical challenges. Free-space cavities typically suffer from limited coupling to optical fibers, which complicates their integration into large-scale quantum networks. Microtoroid and microdisk resonators exhibit ultrahigh quality factors, yet stable and scalable optical interfacing, as well as the integration of multiple devices, remain nontrivial.

Against this backdrop, cavity QED based on optical nanofibers (ONF) has emerged as a distinctive and powerful alternative. An ONF is a silica optical fiber tapered down to a diameter comparable to or smaller than the optical wavelength. In such fibers, a significant fraction of the guided optical mode extends outside the fiber surface in the form of an evanescent field. Atoms positioned in this near-field region experience interactions with the fiber-guided light that are substantially enhanced compared with free space. Moreover, by incorporating fiber Bragg gratings at both ends of the nanofiber waist, one can form a high-*Q* optical cavity that is fully integrated within a fiber-based geometry. This “fiber-inline cavity” architecture naturally provides efficient and low-loss input–output coupling, making it particularly attractive for quantum network applications.

The key advantage of nanofiber cavity QED lies in its unique ability to simultaneously achieve strong atom–photon coupling and seamless compatibility with optical fiber communication. Not only can strong interactions be realized at the single-atom level, but the associated photons can also be extracted into standard optical fibers with minimal loss. This combination directly addresses a central requirement for distributed quantum computing and quantum networks. At a quantum node in a quantum network, a single atom generates, stores, and processes quantum states that are carried by photons through optical fibers to distant nodes, enabling the creation of remote entanglement. Nanofiber cavities provide a particularly natural platform for these tasks, as they merge cavity QED physics with fiber-based quantum interfaces in a single system.

In parallel with cavity-based approaches, another powerful paradigm has emerged in recent years: waveguide quantum electrodynamics (waveguide QED). In this framework, quantum emitters interact with photons propagating in a one-dimensional guided mode, such as that provided by optical fibers, nanophotonic waveguides, or superconducting transmission lines. Because the electromagnetic field is confined transversely while remaining free to propagate along the waveguide, the interaction between an emitter and a traveling photon can be significantly enhanced compared with free space.

Optical nanofibers provide an excellent platform for realizing waveguide QED. Atoms located near the nanofiber surface can couple strongly to the evanescent field of the guided mode, leading to efficient atom–photon interactions in a one-dimensional geometry. In addition, the guided mode of a nanofiber is naturally and efficiently transferred to standard single-mode optical fibers through its adiabatic tapered sections, enabling low-loss interfacing with fiber-based photonic systems. Optical nanofiber platforms therefore offer a unique opportunity to explore both cavity-enhanced and waveguide-mediated regimes.

In this review, we begin by introducing the physical principles underlying optical nanofiber cavity and waveguide QED. We then discuss the key experimental techniques used to realize these systems, the characteristic phenomena that have been observed, and their potential applications in quantum information science.

## Theories and methods

### Cavity QED and waveguide QED

The energy levels of an electron bound in the Coulomb potential of an atomic nucleus are not equally spaced due to the anharmonicity of the Coulomb potential. As a consequence, a single atom can absorb or emit only one photon at a time through an electronic transition, making it an ideal single-photon emitter. Atomic states with long coherence times can also serve as stationary qubits or quantum memories. Furthermore, the anharmonicity of the Coulomb potential gives rise to strong optical nonlinearities at the single-photon level, enabling the implementation of quantum gates for both atomic and photonic qubits. By combining these functionalities, it becomes possible to construct large-scale quantum information processing networks [1–5].

To fully exploit these functionalities, it is crucial to enhance the interaction between atoms and light. Two important frameworks enable such enhancement. One is cavity QED, the physics of atom–photon interactions inside an optical resonator (cavity), where light is confined within a small volume [6]. The other is waveguide QED, the physics of atom–photon interactions in a guided optical mode, where light is tightly confined in the transverse direction while propagating along a waveguide. In this subsection, we review the basic theory and relevant parameters of cavity and waveguide QED.

#### Atom–photon interaction in cavity QED

The resonant absorption cross section of an atom in free space is given by

1 $$\begin{aligned} \sigma = \frac{3\lambda _\textrm{A}^2}{2\pi }, \end{aligned}$$

where $\lambda _\textrm{A}$ denotes the resonant wavelength of the atom. In order to realize efficient atom–photon interactions in free space, the cross-sectional area of the optical beam must be comparable to or smaller than *σ *, which is generally difficult to achieve.

The interaction between atoms and light can instead be strongly enhanced by using an optical cavity. The fundamental Hamiltonian of a cavity QED system consisting of a single two-level atom and a single cavity mode is known as the Jaynes–Cummings Hamiltonian and is given by

2 $$\begin{aligned} \hat{H}_\textrm{JC}&= \hbar \omega _\textrm{A} \hat{\sigma }^+ \hat{\sigma }^- + \hbar \omega _\textrm{C} \hat{a}_\textrm{c}^\dag \hat{a}_\textrm{c} + \hbar g(\hat{\sigma }^+\hat{a}_\textrm{c} + \hat{\sigma }^- \hat{a}_\textrm{c}^\dag ), \end{aligned}$$

where $\hat{\sigma }^+$ and $\hat{\sigma }^-$ are the atomic raising and lowering operators, respectively, and $\hat{a}_\textrm{c}^\dag$ and $\hat{a}_\textrm{c}$ are the photon creation and annihilation operators for the cavity mode. The quantities $\omega _\textrm{A}=2\pi c/\lambda _\textrm{A}$ and $\omega _\textrm{C}$ denote the resonance frequencies of the atom and the cavity mode, respectively, and $\hbar = h/(2\pi )$ is the reduced Planck constant.

The atom–cavity coupling rate is given by

3 $$\begin{aligned} g&= \frac{\mu E_0}{\hbar } u(\textbf{r}), \end{aligned}$$

where *μ * is the electric dipole moment of the atomic transition and $u(\textbf{r})$ is the dimensionless cavity mode function normalized to the maximum electric field amplitude. The vacuum electric-field amplitude is given by

4 $$\begin{aligned} E_0=\sqrt{\frac{\hbar \omega _\textrm{C}}{2\varepsilon _0 V}}, \end{aligned}$$

where $\varepsilon _0$ is the vacuum permittivity and *V* is the cavity mode volume. Furthermore, by defining the *effective* mode volume at the atomic position $\textbf{r}$ as

5 $$\begin{aligned} V_\textrm{eff}(\textbf{r}) =\frac{\int \varepsilon (\textbf{r}')|\textbf{E}(\textbf{r}')|^2\,dV}{\varepsilon (\textbf{r})|\textbf{E}(\textbf{r})|^2}, \end{aligned}$$

the coupling rate can be written as

6 $$\begin{aligned} g&= \mu \sqrt{\frac{\omega _\textrm{C}}{2\hbar \varepsilon _0 V_\textrm{eff}(\textbf{r})}}, \end{aligned}$$

which explicitly expresses the spatial dependence of the coupling strength through the effective mode volume. In addition, for a single-pass propagating mode (in the absence of cavity mirrors), the effective mode area at position $\textbf{r}$ can be defined as

7 $$\begin{aligned} A_\textrm{eff}(\textbf{r}) =\frac{\int \varepsilon (\mathbf {r'})|\textbf{E}(\mathbf {r'})|^2\,dA}{\varepsilon (\textbf{r})|\textbf{E}(\textbf{r})|^2}. \end{aligned}$$

For a cavity of length *L*, the effective mode volume can be related to the effective mode area as

8 $$\begin{aligned} V_\textrm{eff}(\textbf{r}) = A_\textrm{eff}(\textbf{r})L \end{aligned}$$

for a ring cavity, while for a Fabry–Pérot cavity

9 $$\begin{aligned} V_\textrm{eff}(\textbf{r}) = A_\textrm{eff}(\textbf{r})L/2, \end{aligned}$$

where the factor of 1/2 arises from the standing-wave nature of the cavity field.

The eigenstates and corresponding eigenenergies of the Jaynes–Cummings Hamiltonian $\hat{H}_\textrm{JC}$ are obtained by diagonalizing Eq. (2). The state of the coupled atom–cavity system can be written as

10 $$\begin{aligned} |i,n\rangle = |i\rangle _\textrm{A} \otimes |n\rangle _\textrm{C}, \end{aligned}$$

where $|i\rangle _\textrm{A}$ and $|n\rangle _\textrm{C}$ denote the atomic and cavity states, respectively, and *n* is the number of photons in the cavity. The ground state and its eigenenergy are the same as in the absence of interaction and are given by

11 $$\begin{aligned} |\Psi ^{(0)}\rangle&= |g,0\rangle , \end{aligned}$$

12 $$\begin{aligned} E^{(0)}&= 0 . \end{aligned}$$

The excited states are superpositions of *|g,n〉 * and *|e,n-1〉 *. In particular, for the resonant case $\omega _\textrm{A}=\omega _\textrm{C}$, the eigenstates are

13 $$\begin{aligned} |\Psi _{+}^{(n)}\rangle&= \frac{1}{\sqrt{2}} |e,n-1\rangle + \frac{1}{\sqrt{2}} |g,n\rangle , \end{aligned}$$

14 $$\begin{aligned} |\Psi _{-}^{(n)}\rangle&= -\frac{1}{\sqrt{2}} |e,n-1\rangle + \frac{1}{\sqrt{2}} |g,n\rangle , \end{aligned}$$

with corresponding eigenenergies

15 $$\begin{aligned} E_{\pm }^{(n)} = n\hbar \omega _\textrm{C} \pm \sqrt{n}\,\hbar g . \end{aligned}$$

The splitting of the first excited-state doublet (*n=1*), *2ℏ g*, is known as the vacuum Rabi splitting.

#### Dissipations in cavity QED

A cavity QED system involves two fundamental dissipative processes: spontaneous emission of the atom and photon leakage from the cavity. An atom in the excited state can decay to the ground state by spontaneously emitting a photon into free space. The corresponding *energy* decay rate (“Einstein A coefficient”) is given by

16 $$\begin{aligned} \Gamma = \frac{1}{4\pi \varepsilon _0 }\frac{4 \omega _\textrm{A}^3 \mu ^2}{3 \hbar c^3}. \end{aligned}$$

The corresponding *amplitude* decay rate is

17 $$\begin{aligned} \gamma = \frac{\Gamma }{2} . \end{aligned}$$

Photons leak out of the cavity due to external and internal losses. External loss corresponds to photons escaping from the cavity through the finite transmission of the cavity mirrors, while internal loss arises from absorption and scattering at the mirrors or from propagation losses inside the cavity. The corresponding electric-field decay rates for the external and internal losses are given by

18 $$\begin{aligned} \kappa _{j, \mathrm ex}&= \frac{c}{4L}T_j, \end{aligned}$$

19 $$\begin{aligned} \kappa _\textrm{in}&= \frac{c}{4L}\alpha _\textrm{loss}, \end{aligned}$$

where $T_j$ and $\alpha _\textrm{loss}$ denote the transmission of mirror *j (=1,2)* and the internal round-trip loss, respectively. The total electric-field decay rate is therefore

20 $$\begin{aligned} \kappa = \kappa _{1, \mathrm ex} + \kappa _{2, \mathrm ex} + \kappa _\textrm{in} = \frac{c}{4L}\alpha _\textrm{total}, \end{aligned}$$

where $\alpha _\textrm{total}=T_1+T_2+\alpha _\textrm{loss}$ represents the total round-trip loss. The total *energy* decay rate *2κ * is equal to the full width at half maximum (FWHM) of the cavity resonance,

21 $$\begin{aligned} \Delta _\textrm{FWHM} = 2\kappa , \end{aligned}$$

and is also the inverse of the photon lifetime in the cavity,

22 $$\begin{aligned} \tau = \frac{1}{2\kappa }. \end{aligned}$$

The escape efficiency through mirror *j* is given by

23 $$\begin{aligned} \eta _{j, \mathrm esc} = \frac{\kappa _{j, \mathrm ex}}{\kappa }, \end{aligned}$$

which corresponds to the probability that a photon exits the cavity to the output mode associated with mirror *j*.

Two commonly used figures of merit characterizing an optical cavity are the finesse and the quality factor. The finesse $\mathcal {F}$ is defined as

24 $$\begin{aligned} \mathcal {F} = \frac{\Delta _\textrm{FSR}}{\Delta _\textrm{FWHM}} = \frac{2\pi }{\alpha _\textrm{total}}, \end{aligned}$$

where $\Delta _\textrm{FSR}$ is the free spectral range. The finesse depends only on the total round-trip loss $\alpha _\textrm{total}$. The quantity $\mathcal {F}/(2\pi )=1/\alpha _\textrm{total}$ represents the average number of round trips a photon makes inside the cavity before escaping.

The cavity quality factor *Q* is defined as

25 $$\begin{aligned} Q = \frac{\omega _\textrm{C}}{\Delta _\textrm{FWHM}} = \frac{4\omega _\textrm{C}L}{c(T+\alpha )}, \end{aligned}$$

which depends not only on the total round-trip loss but also on the resonance angular frequency $\omega _\textrm{C}$ and the cavity length *L*. The quantity $Q/(2\pi )=\omega _\textrm{C}\tau /(2\pi )$ represents the number of optical oscillations of the electric field during the photon lifetime in the cavity.

#### Cooperativity parameter in cavity QED

Cavity QED exhibits two qualitatively different regimes: the strong-coupling regime, which satisfies $g \gg (\kappa ,\gamma ),$ and the Purcell regime, which satisfies $\kappa \gg g^2/\kappa \gg \gamma$. In the strong-coupling regime, the dynamics of the system are dominated by coherent and reversible interactions between the atom and the cavity field. In the time domain, vacuum Rabi oscillations can be observed, while in the frequency domain the system exhibits vacuum Rabi splitting. In contrast, in the Purcell regime the cavity decay is the fastest process, so that a photon emitted by the atom escapes from the cavity before it can be reabsorbed. In this regime it is useful to introduce the cooperativity parameter

26 $$\begin{aligned} C&= \frac{g^2}{2\kappa \gamma } \left( = \frac{g^2}{\kappa \Gamma } \right) , \end{aligned}$$

which quantifies the strength of the atom–cavity interaction relative to the dissipation rates. An atom in the excited state then decays to the ground state at a rate *(1+2C)Γ *, which is faster than the free-space decay rate *Γ *. This enhancement arises because the spontaneous emission into the cavity mode is increased by a factor 2*C* compared with that into free space, which is known as the Purcell effect. The enhancement factor (Purcell factor) can also be derived from Fermi’s golden rule [7]:

27 $$\begin{aligned} 2C = \frac{3}{4\pi ^2}\frac{Q}{V}\lambda _\textrm{A}^3, \end{aligned}$$

which is consistent with Eq. (26).

Furthermore, from Eqs. (3), (16), and (20), the cooperativity can be written as

28 $$\begin{aligned} C = \frac{1}{2\,\alpha _\textrm{total}\,\tilde{A}_\textrm{eff}}, \end{aligned}$$

where $\tilde{A}_\textrm{eff}$ is the effective mode area normalized by the resonant absorption cross section *σ * of the atom in free space,

29 $$\begin{aligned} \tilde{A}_\textrm{eff} = \frac{A_\textrm{eff}}{\sigma } = \frac{2\pi }{3}\frac{A_\textrm{eff}}{\lambda _\textrm{A}^2}. \end{aligned}$$

It is also useful to define the internal cooperativity, which is determined by the internal loss,

30 $$\begin{aligned} C_\textrm{int} = \frac{1}{2\,\alpha _\textrm{loss}\,\tilde{A}_\textrm{eff}} . \end{aligned}$$

#### Atom–photon interaction in waveguide QED

While the coupling rate between the cavity mode and the atom is given by Eq. (3), the coupling rate between a guided mode of a waveguide and an atom is given by [8]

31 $$\begin{aligned} g_\textrm{W} = \mu \sqrt{\frac{\omega _\textrm{W}}{4\pi \varepsilon _0 \hbar A_\textrm{eff}}}. \end{aligned}$$

The decay rate of the atom into the waveguide mode is given by

32 $$\begin{aligned} \Gamma _\textrm{1D} = \frac{4\pi g_\textrm{W}^2}{v_g} = \frac{1}{2}\frac{c}{v_g}\frac{\sigma }{A_\textrm{eff}}\Gamma , \end{aligned}$$

where $v_g$ is the group velocity. The coupling efficiency between the atom and the waveguide, i.e. the fraction of photons emitted by the atom that couple to the guided mode, is given by

33 $$\begin{aligned} \beta = \frac{\Gamma _\textrm{1D}}{\Gamma _\textrm{1D} + \Gamma ^\prime }, \end{aligned}$$

where $\Gamma ^\prime$ denotes the decay rate into all modes other than the waveguide mode. For ONFs, $\Gamma ^\prime \approx \Gamma$, which leads to

34 $$\begin{aligned} \beta \approx \frac{\Gamma _\textrm{1D}}{\Gamma _\textrm{1D} + \Gamma } = \frac{1}{2}\frac{c}{v_g}\frac{\sigma }{A_\textrm{eff}} . \end{aligned}$$

### Quantum information applications

By strongly coupling quantum systems like individual atoms to a well-defined optical mode, cavity QED enables the controlled exchange of quantum information between stationary (atomic) and flying (photonic) degrees of freedom. This capability makes cavity QED systems a central building block for quantum computing, quantum communication, and quantum networking applications [2, 9, 10].

#### Photon-mediated quantum gates

**Fig. 1:**
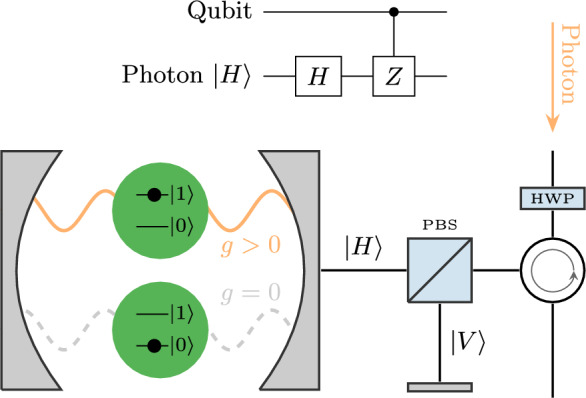
An atom with qubit-state dependent coupling to the cavity enables the controlled exchange of quantum information between atomic and photonic degrees of freedom. Together with a half-wave plate (HWP) acting as a photonic Hadamard gate, a polarizing beam-splitter (PBS), and an optical circulator, this setup constitutes an atom–photon controlled-*Z* gate

The simplest form of photon-mediated quantum gates in cavity QED systems works by selectively shifting the cavity resonance frequency depending on some internal degree of freedom of the coupled quantum systems, for example the qubit basis states $\vert 0\rangle$ and $\vert 1\rangle$ in an atom. A photon impinging onto the cavity then experiences a state-dependent phase shift of its wave function [11, 12]. A basic setup for exchanging quantum information between atomic and photonic degrees of freedom is sketched in Fig. 1. It constitutes an atom–photon controlled phase-flip (CPF) or equivalently controlled-Z (CZ) gate. Together with the local and remote atom–atom variants derived from it, they are also known as Duan–Kimble gates [13, 14]. More recently, other schemes involving virtual photon exchanges [15], more complex level structures for error protection [16], or auxiliary atoms for heralding [17] have been proposed. Likewise, the mechanism works for performing photon–photon CPF [18] and $\sqrt{\textrm{SWAP}}$ [19] gates, as well as multiqubit CPF gates if several atoms are coupled to the cavity [20–22]. Similarly to other quantum computing architectures, such as superconducting qubits or trapped ions, photon-mediated CZ gates are biased towards phase-flip errors. In error correction schemes like surface codes, this can result in relaxed requirements for the necessary error thresholds [23, 24].

For single-photon pulses of sufficient length with negligible pulse delay and distortion, the final state of the combined system can be written as [25]

35 $$\begin{aligned} \vert \psi \rangle= & \frac{1}{\sqrt{2}}[r(g=0)\alpha \vert H0\rangle + r(g>0)\beta \vert H1\rangle +\alpha \vert V0\rangle \nonumber \\ & + \beta \vert V1\rangle ] =: \hat{U}\vert \psi _0\rangle \;, \end{aligned}$$

where the cavity reflection coefficient for a cavity with decay rate $\kappa _r$ for the in- and out-coupling mirror and a photon in the long-pulse limit with a narrow frequency band centered around $\omega _p$ reads

36 $$\begin{aligned} r(\omega _p) = 1 + \frac{2\kappa _r}{i\left( \omega _p - \omega _c\right) - \kappa + \frac{g^2}{i\left( \omega _p - \omega _a\right) - \gamma }} \;. \end{aligned}$$

Here, $\kappa \ge \kappa _r$ denotes the total decay rate of the cavity, which may include photon loss via transmission or internal scattering. The coupling strength between the light field and the atom is denoted by *g* and the excited state at an energy of $\hbar \omega _a$ above the upper qubit state $\vert 1\rangle$ decays at a rate of *γ *. For suitable system parameters we have *r(g=0)→ -1* and *r(g>0)→ 1* and the gate unitary $\hat{U}=\exp \left( i\pi \vert H0\rangle \langle H0\vert \right)$ is performed.

For reducing gate operation times, photon pulses with finite durations need to be considered. There, the state-dependent phase shift also results in state-dependent pulse delays and distortions, affecting gate fidelity. Furthermore, including potential pulse delays or photon loss in the $\vert V\rangle$ component beam splitter arm via a complex-valued duration $\tau _\textrm{delay}$ according to $\vert p\rangle \sim r(\omega )\vert H\rangle + e^{-i\omega \tau _\textrm{delay}}\vert V\rangle$ offers another way to optimize gate fidelities at the cost of reduced success probabilities [26].

Using the same photon to perform two atom–photon CZ gates in sequence on two atomic qubits located in distant cavities, a remote atom–atom controlled-Z gate can be achieved [27, 28]. Similarly, if two atoms are coupled to the same cavity, a local atom–atom CZ gate can be performed on them via the photon-mediated gate mechanism [29]. Assuming equal coupling strengths $g_1 = g_2 =:g$ for both atoms, the reflection coefficient in Eq. (36) is modified according to *r(g)→ r(Ng)* and tracing out the photon after a successful reflection, the unitary

37 $$\begin{aligned} \hat{U} = \operatorname {diag}\left\{ r(N=0),r(N=1),r(N=1),r(N=2)\right\} \end{aligned}$$

is applied to the two-qubit atomic state. Depending on the photon–cavity detuning, different unitary operations on the atomic qubits can be realized. A photon on resonance with the bare cavity frequency, $\omega _p=\omega _c$, results in the unitary $\hat{U}=\exp \left( i\pi \vert 00\rangle \langle 00\vert \right)$. On the other hand, adjusting the photon frequency to the Rabi splitting according to $\omega _p - \omega _c \sim \sqrt{2}g$ yields the conventional CZ unitary $\hat{U}=\exp \left( i\pi \vert 11\rangle \langle 11\vert \right)$.

In general, average fidelities for evaluating quantum gate performance need to be calculated numerically. However, an analytical approximation of the local gate fidelity reading [25]

38 $$\begin{aligned} F_\textrm{local} = \frac{1}{4}\frac{\vert r(0) - 2r(1) - r(2)\vert ^2}{\vert r(0)\vert ^2 + 2\vert r(1)\vert ^2 + \vert r(2)\vert ^2} \end{aligned}$$

provides insight for optimizing the gate performance. The reflection coefficients, assuming resonance $\omega _p = \omega _c = \omega _a,$ simplify to $r(N) = 1 - 2\kappa _r/(\kappa + Ng^2/\gamma )$.

The condition *|r(0)|=|r(1)|* leads to an expression for the coupling strength $g_*$ and the loss ratio $\kappa _r/\kappa$ at which the fidelity becomes approximately maximum according to

39 $$\begin{aligned} C^* = \frac{g^2_*}{\gamma \kappa } = \frac{\kappa _r/\kappa }{1 - \kappa _r/\kappa } - 1 \;, \end{aligned}$$

where we have defined the cooperativity $C^*$ and assume that $\kappa _r/\kappa >1/2$. Note that $C^* = 2C$ compared to conventional definitions of cooperativity *C*, see e.g.,  Eq. (26) or Ref. [2].

The fidelity exhibits a maximum as a result of photon loss in the cavity. Effects such as transmission and mirror scattering losses reduce the absolute value of the reflection coefficient in the non-coupling state compared to its ideal value of minus one, *|r(g=0)|<1*. At the maximum, the value of $F_\textrm{max}$ depends on the ratio $\kappa _r/\kappa$ according to

40 $$\begin{aligned} F_\textrm{max} = 1 - \frac{3\left( 1 - \frac{\kappa _r}{\kappa }\right) ^2}{4\left[ 7\left( \frac{\kappa _r}{\kappa }\right) ^2 - 4\frac{\kappa _r}{\kappa } + 1\right] } \;. \end{aligned}$$

**Fig. 2:**
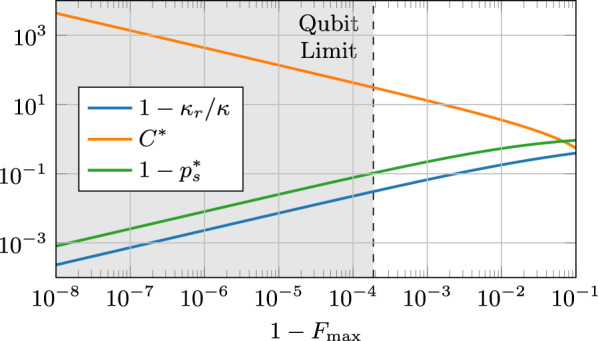
Cavity loss ratio $1-\kappa _r/\kappa$ and cooperativity $C^*$ required for reaching infidelity $1 - F_\textrm{max}$ according to Eq. (39) together with gate error probability $1-p_s^*$. The finite qubit level splitting $\omega _q$ limits the maximum fidelity according to $1 - F_\textrm{max}\sim 2\gamma /\omega _q$. The shaded area indicates the inaccessible region for cesium qubits with $1 - F_\textrm{max} > 1.88\times 10^{-4}$. Reprinted with permission from Ref. [25]. © Optica Publishing Group

It can also be expressed as a function of cooperativity by replacing $\kappa _r/\kappa = (C^*+1)/(C^*+2)$. The loss ratio and cooperativity required for obtaining a certain value of $F_\textrm{max}$ together with the corresponding error probability $1 - p_s^*$ are plotted in Fig. 2.

The fidelity is further globally limited by the energy difference $\hbar \omega _q$ between the computational basis states $\vert 0\rangle$ and $\vert 1\rangle$. For example, for a qubit defined through the hyperfine clock states of cesium, the state $\vert 0\rangle$ is effectively not coupled to the cavity due to the large but finite splitting $\omega _q/(2\pi)\sim 9.2$ GHz [30]. However, the coupling strengths required to reach fidelities close to one according to Eq. (39) can become so large that the cavity resonance frequency is shifted to a regime in which the otherwise sufficiently detuned $\vert 0\rangle$ state also significantly interacts with the cavity mode. The finite qubit splitting therefore constrains the fidelity in leading order to $1 - F_\textrm{max}\sim 2\gamma /\omega _q$. Generally, the lower qubit state physically couples to the cavity via a separate excited state $\vert e'\rangle$ with modified coupling strengths and level splitting. For cesium qubits, this translates to a maximum fidelity of $F_\textrm{max} = 0.9998$ that is reached for a cavity with a loss ratio of $\kappa _r/\kappa = 0.9696$ and a cooperativity of $C^* = 30.86$ as indicated by the shaded area in Fig. 2.

#### Single-photon sources

**Fig. 3:**
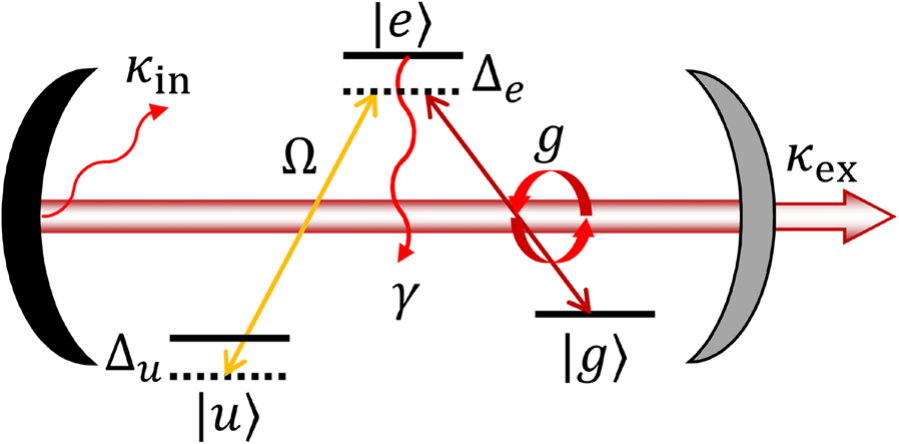
Cavity QED setup for single-photon generation with a *Λ *-type quantum emitter. In the case of vSTIRAP, starting in $\vert u\rangle$ the external pump laser strength *Ω (t)* is gradually increased such that the excited state $\vert e\rangle$ remains unpopulated while the emitter state is transferred to $\vert g\rangle$, creating a cavity excitation in the process. Reprinted with permission from Ref. [31]. © American Physical Society

Quantum information technologies involving networking, communication, cryptography, or computing architectures such as the cavity-based quantum gates all require quantum light sources that can produce single photons with deterministic properties on demand. Cavity QED setups are ideal single-photon sources for this purpose [32, 33]. The photon generation process generally works by exciting the quantum emitter and ensuring that it predominantly decays into the cavity mode and not into free space. The single cavity excitation then further decays through one of the cavity mirrors and the photon can be used [34, 35]. Importantly, the single-photon generation process is reversible and the cavity QED system can also act as a single-photon receiver or quantum memory [36–38].

In the bad-cavity regime, decay into the cavity mode is enforced through the Purcell effect. It enhances the decay into the cavity mode by a factor of 2*C* compared to free-space emission. Most state-of-the-art experiments use a vacuum-stimulated Raman adiabatic passage (vSTIRAP) scheme for quantum emitters with a *Λ *-type three-level structure instead, as shown in Fig. 3, where the coupled system remains in a dark state without populating the excited state throughout the process [39]. It offers the additional benefit of allowing nearly arbitrary control over the output photon shape [40, 41]. The total photon generation efficiency comprises a trade-off between the efficiency of generating an excitation inside the cavity and out-coupling it through the mirrors. In the long-pulse or adiabatic limit, it is given by [31]

41 $$\begin{aligned} \eta _\textrm{total} = \frac{\kappa _\textrm{ex}}{\kappa }\frac{2C}{1 + 2C} \;. \end{aligned}$$

Increasing the out-coupling efficiency $\eta _\textrm{ex} = \kappa _\textrm{ex}/\kappa$ by increasing $\kappa _\textrm{ex}$ results in a decreased cooperativity *C* and therefore decreased generation efficiency $\eta _\textrm{int} = 2C/(1 + 2C)$. The overall efficiency can be optimized by adjusting the mirror decay rate $\kappa _\textrm{ex}$ to the internal cavity loss rate $\kappa _\textrm{in}$ according to $\kappa _\textrm{ex} = \kappa _\textrm{in}\sqrt{1 + 2C_\textrm{in}},$ where the internal cooperativity is defined as $C_\textrm{in} = g^2/2\kappa _\textrm{in}\gamma$ [31].

Using a Gaussian wave-packet model, the success probability of generating a photon and its width can be optimized for finite-time pulses in the non-adiabatic regime as well, for coupling strengths ranging from the Purcell regime to the strong-coupling regime [42]. If a quantum emitter with four levels and a second excited state is used instead of the three-level *Λ *-scheme, the purity of the generated photons can be increased by suppressing re-excitation [43].

### Optical nanofibers

Optical fibers can be modeled as dielectric step-index cylinders, consisting of a core with radius $a_0$ and refractive index $n_2$, surrounded by cladding with refractive index $n_1$. Since the refractive index of the core is larger than that of the cladding, $n_2 > n_1$, light propagating through the fiber is confined to the core by total internal reflection. This principle is also described as the cladding guiding light through the core [44, 45].

#### Adiabatic tapers

**Fig. 4:**
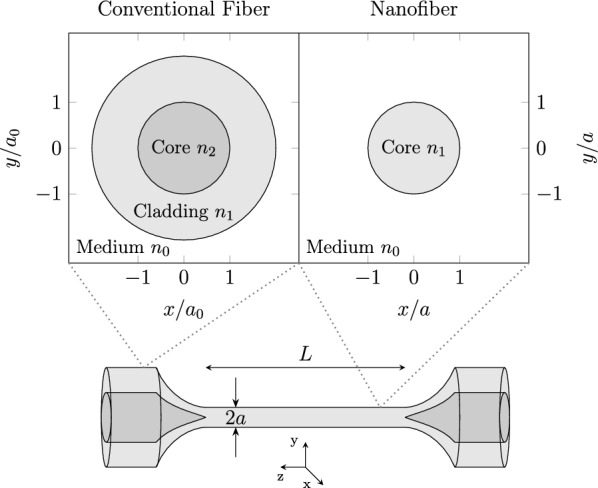
Sketch of the step-index profile of a conventional fiber consisting of core and cladding in a surrounding medium with respective refractive indices of $n_2> n_1 > n_0$ tapering into a nanofiber portion of length *L* and radius *a*. The original cladding forms the nanofiber core with refractive index $n_1$. Proportions are not to scale. Conventional fibers typically have a diameter of 125 *μ *m including cladding, with a core radius on the order of $a_0\sim 3$ *μ *m. The taper region usually spans several centimeters, ending in a nanofiber radius of *a≲ 300* nm [49]

Nanofibers are produced by thinning or tapering conventional single-mode optical fibers to diameters smaller than the wavelength of their guided mode. The fiber cladding is replaced by the surrounding medium, e.g., air or vacuum, with refractive index $n_0$, and the original cladding forms the new fiber core with radius *a*. Light is then purely guided by the surrounding medium and the refractive index difference between core and cladding is on the order of $n_1 - n_0\approx 0.5$, compared to the so-called weakly-guided regime of commercial fibers that typically have $n_2-n_1\ll 1$ [46]. As a result, a significant part of the transmitted light propagates as an evanescent field outside the fiber and can be used for, e.g., interfacing single atoms with the fiber [47, 48]. Figure 4 shows a sketch of a conventional fiber with a step-index refractive index profile tapering into a nanofiber section.

In order to achieve high transmission through the fiber, the taper has to be designed in such a way that coupling of the fundamental mode in a single-mode fiber to higher-order modes as a result of the varying fiber diameter is minimized [49]. This is mainly achieved by limiting the tapering angle, i.e. the change of the fiber radius along the cylinder direction $\partial r/\partial z$. One commonly suggested criterion for achieving such adiabatic coupling is given by [50]

42 $$\begin{aligned} \left| \frac{\partial r}{\partial z}\right| \le \frac{r}{2\pi }\left[ \beta _1(z) - \beta _2(z)\right] \;, \end{aligned}$$

where *r* is the local fiber radius at position *z* and $\beta _1(z)$ and $\beta _2(z)$ are the local propagation constants of the fundamental mode HE$_{11}$ and first excited mode HE$_{12}$ respectively.

#### Guided modes

Due to the symmetry of the system, for guided light modes propagating along the *z*-direction of the cylinder, both the electric and magnetic fields $\psi \in \lbrace E,H\rbrace$ take the form of $\mathbf {\psi }(\textbf{r},t) = \mathbf {\psi }(r,\varphi )\exp \left[ i\left( \omega t - \beta z\right) \right]$. The radial and azimuthal field components $\psi _r$ and $\psi _\varphi$ can be determined by expressing Maxwell’s equations as a function of the axial component $\psi _z$, which in turn is calculated from the wave equation [51]:

43 $$\begin{aligned} \left[ \frac{\partial ^2}{\partial r^2} + \frac{1}{r}\frac{\partial }{\partial r} + \left( (n(r)k)^2 - \beta ^2 - \frac{l^2}{r^2}\right) \right] \psi _z = 0 \;, \end{aligned}$$

where we have further separated the field into radial and azimuthal contributions according to $\psi _z(r,\varphi )$ $= \psi _z(r)\exp (\pm il\varphi )$ with integer *l* and where $k=2\pi /\lambda$ is the wave number of the input light in vacuum. From the general solution of Bessel’s differential equation (43) and its asymptotic behavior for *r→ 0* and $r\rightarrow \infty$, we can conclude that the fields must be of the form $\psi _z(r) \sim K_l(wr/a)$ for $w^2 = a^2(\beta ^2 - n_0^2k^2) > 0$ outside the fiber (*r>a*) and $\psi _z(r) \sim J_l(ur/a)$ for $u^2 = a^2(n_1^2k^2 - \beta ^2) > 0$ inside the fiber (*r<a*), where $J_l(x)$ and $K_l(x)$ are the Bessel functions of the first kind and modified Bessel functions of the second kind respectively.

The required positivity of the effective wave numbers *u* and *w* leads to the necessary condition

44 $$\begin{aligned} n_0< \beta /k < n_1 \end{aligned}$$

for the existence of guided modes. The tangential field components $\psi _\varphi$ and $\psi _z$ need to be continuous at the fiber boundary *r = a*. The resulting set of four equations for the field coefficients has a non-trivial solution if their determinant vanishes. This leads to the fiber eigenvalue equation [46],

45 $$\begin{aligned} & l^2\left( \frac{n_1^2}{u^2} + \frac{n_0^2}{w^2}\right) \left( \frac{1}{u^2} + \frac{1}{w^2}\right) \nonumber \\ & \quad = \left( n_1^2\frac{J_l'(u)}{uJ_l(u)} + n_0^2\frac{K_l'(w)}{wK_l(w)}\right) \left( \frac{J_l'(u)}{uJ_l(u)} + \frac{K_l'(w)}{wK_l(w)}\right) , \end{aligned}$$

for the effective wave numbers. Guided modes are solutions of Eq. (45) lying on the first quadrant of the circle

46 $$\begin{aligned} u^2 + w^2 = a^2k^2\left( n_1^2 - n_0^2\right) \equiv v^2 \;, \end{aligned}$$

where the quantity *v* is also known as the fiber volume. The propagation constant *β * of the guided mode is then finally calculated from the relation [46, 52]

47 $$\begin{aligned} \frac{\beta ^2}{k^2} = \left( \frac{n_1^2}{u^2} + \frac{n_0^2}{w^2}\right) \left( \frac{1}{u^2} + \frac{1}{w^2}\right) ^{-1}, \end{aligned}$$

or directly from the definitions of *u* and *w*.

**Fig. 5:**
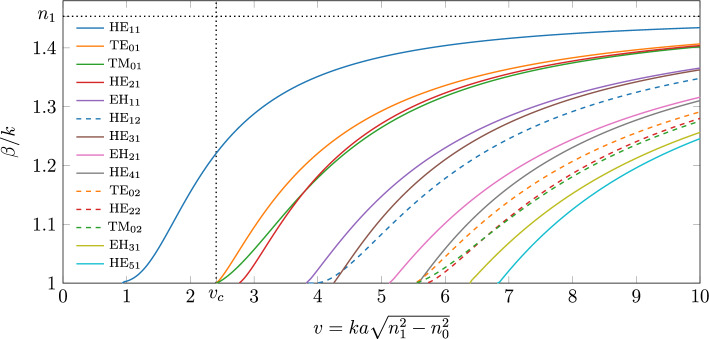
Propagation constants $\beta _{lm}$ of guided modes according to Eq. (45) as a function of fiber volume *v* and a nanofiber with $n_1 = 1.4537$ and $n_0=1.$ See text for an explanation of the mode nomenclature. Below a fiber volume of $v_c\approx 2.405$ the single-mode condition is fulfilled and only the fundamental HE$_{11}$ mode propagates through the fiber. Modes $\beta _{l2}$ are shown as dashed lines

In general there are several solutions fulfilling Eqs. (45) and (46) for a given value of *l*. The resulting guided modes are typically labeled $\beta _{ml}$ for the *m*-th solution of order *l*. For *l = 0*, the guided modes are also referred to as TM$_{0m}$ ($H_z\equiv 0$) or TE$_{0m}$ ($E_z\equiv 0$) modes. For *l\ge 1*, they are hybrid modes labeled as EH$_{lm}$ or HE$_{lm}$, corresponding to solutions in the positive and negative branch of Eq. (45), arising from its quadratic form. Physically, in EH modes the axial magnetic field $H_z$ tends to be dominant, while in HE modes the axial electric field $E_z$ is relatively strong. If $n_0\approx n_1$, Eq. (45) can be simplified and the resulting approximate solutions are referred to as linearly polarized or LP modes [51]. Figure 5 shows the normalized fiber propagation constants $\beta _{lm}/k$ for a nanofiber with $n_1 = 1.4537$ and $n_0=1$. If the fiber volume is smaller than the cutoff value $v\le v_c \approx 2.4048$, determined from $J_0(v_c) = 0$, the fiber supports only a single guided mode. For light at a wavelength of *λ = 852.3* nm close to the D2 line of cesium, this corresponds to a nanofiber radius of 309 nm.

Using these propagation constants, the electric field of a specific guided mode reads [51], for *r>a*,

48 $$\begin{aligned} & \textbf{E}\left( \textbf{r},t\right) = \begin{pmatrix} E_r\\ E_\varphi \\ E_z \end{pmatrix}=A\frac{J_l(u)}{K_l(w)}\nonumber \\ & \quad \begin{pmatrix} i\frac{a\beta }{w}\left[ K_l'(wr/a) - \frac{lsa}{wr}K_l(wr/a)\right] \\ -\frac{a\beta }{w}\left[ \frac{la}{wr}K_l(wr/a) + sK_l'(wr/a)\right] \\ K_l(wr/a) \end{pmatrix}e^{i\left( \omega t \pm l\varphi -\beta z\right) }, \end{aligned}$$

and for *r<a* we have

49 $$\begin{aligned} & \textbf{E}\left( \textbf{r},t\right) = \begin{pmatrix} E_r\\ E_\varphi \\ E_z \end{pmatrix} \nonumber \\ & \quad = A \begin{pmatrix} -i\frac{a\beta }{u}\left[ J_l'(ur/a) - \frac{lsa}{ur}J_l(ur/a)\right] \\ \frac{a\beta }{u}\left[ \frac{la}{ur}J_l(ur/a) - sJ_l'(ur/a)\right] \\ J_l(ur/a) \end{pmatrix}e^{i\left( \omega t \pm l\varphi -\beta z\right) } \;, \end{aligned}$$

where we have introduced the dimensionless quantity *s* as an abbreviation for

50 $$\begin{aligned} s = l\left( \frac{1}{u^2} + \frac{1}{w^2}\right) \left( \frac{J_l'(u)}{uJ_l(u)} + \frac{K_l'(w)}{wK_l(w)}\right) ^{-1} \;. \end{aligned}$$

The constant *A* depends on the power of the propagating field.

**Fig. 6:**
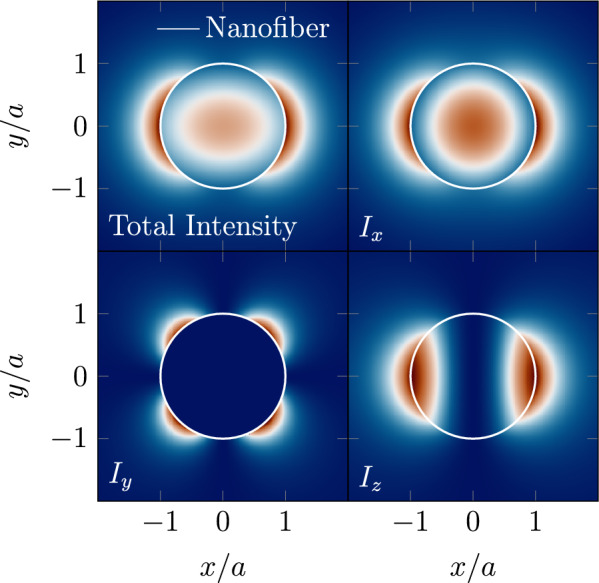
Intensity distribution of the fundamental HE$_{11}$ mode at *z = 0* in a nanofiber with radius *a = 200* nm, refractive index $n_1 =1.4537$ and linearly polarized input light at a wavelength of *λ = 852.3* nm. The intensity in each panel is normalized to its respective maximum value. The guided mode is quasi-linearly polarized and the total intensity has a dominant contribution from the field component along the polarization direction, e.g., *x* as shown here

For scientific applications such as cavity or waveguide QED experiments, single-mode fibers supporting only the fundamental fiber mode HE$_{11}$ are normally used. Figure 6 shows the intensity distribution of this fundamental mode for a nanofiber with radius *a = 200* nm, refractive index $n_1 =1.4537$, and linearly polarized light with a wavelength of *λ = 852.3* nm. The linearly polarized input results in a quasi-linearly polarized guided mode consisting of an equal superposition of left- and right-handed circularly polarized modes, i.e. the *± l* waves in Eqs. (48) and (49) [48].

The single-mode cutoff condition can also be understood as a lower limit for the wavelength of the guided light at a fixed fiber radius. In fact, in many of the experiments described in the following sections, such as the two-color evanescent field trap in Sect. 2.4, light of different wavelengths is sent through the same optical nanofiber simultaneously, fulfilling the single-mode condition for all wavelengths involved. The evanescent fields of guided modes decay on a characteristic length scale *Λ = 1/w* that is proportional to their wavelengths and inversely proportional to the fiber radius [53]. Therefore, especially for the two-color trap, choosing the wavelengths and the fiber radius requires careful consideration for balancing the contributions from each wavelength to the total evanescent field and for achieving the desired potential. The retro-reflection-based side-illumination traps presented in the latter half of Sect. 2.4 provide one way of circumventing this.

### Trapping atoms near an optical nanofiber

Efficient quantum interfaces can be realized by confining quantum emitters—such as atoms—within the evanescent field of an ONF. The central challenge is to position an emitter close enough to the ONF surface in order to interact with the evanescent field. While solid-state quantum emitters can be deposited directly onto the ONF surface, this method cannot be employed to interface neutral atoms with an ONF. Atom–nanofiber interfaces have been demonstrated by overlapping a cold-atom cloud with an ONF [54], and while single atoms have been coupled to ONFs using this method, the resulting single-atom occupancy is inherently probabilistic [55].

Control over both the number and the position of atoms interfaced with the ONF is a critical requirement for realizing a functional nanofiber-based quantum node. Precise spatial localization ensures that each atom experiences a well-defined coupling rate to the guided mode, which is essential for achieving stable and reproducible light–matter interactions. Achieving deterministic, reproducible atom loading and stable confinement at the ONF surface therefore remains a central technological challenge on the path toward scalable fiber-integrated quantum architectures.

To achieve deterministic atom–nanofiber coupling, optical dipole trapping is employed. The optical potential experienced by a cesium atom has been formulated in Refs. [53, 56]. For a permanent dipole in an electric field *E*, the potential energy is given as $U_E = -d\cdot E$, where *d* is the dipole moment and *E* is the electric field, given by

51 $$\begin{aligned} E = \frac{1}{2} \left[ E_0 e^{(-iwt)}+E_0^* e^{(iwt)}\right] , \end{aligned}$$

where $E_0$ is the electric field amplitude. For an atom with an induced dipole, the dipole moment can be written as $d_\textrm{ind} = \alpha E$, where *α * is the induced atomic polarizability. The potential energy $U_E$ for an induced dipole is then given as

52 $$\begin{aligned} U_E = - \int\limits_0^E d_\textrm{ind} dE. \end{aligned}$$

The time averaged potential, $\langle U_E \rangle$ can be derived as

53 $$\begin{aligned} \langle U_E \rangle = - \frac{1}{4} \alpha |E_0|^2. \end{aligned}$$

An atom placed in the evanescent field of an ONF experiences an attractive surface potential arising from its interaction with electromagnetic vacuum fluctuations modified by the nearby dielectric surface. This is known as the Casimir-Polder surface interaction, with the potential experienced by alkali atoms near an ONF described in Refs. [57, 58]. The corresponding potential, $V_{\textrm{CP}}$, depends strongly on the distance *d* between the atom and the dielectric surface. In the short-distance regime $d \ll \lambda /(2\pi )$, the Casimir-Polder potential reduces to the nonretarded van der Waals form, which scales as $V_{\textrm{CP}} \propto -1/d^3$ [58]. A conventional approach to controlling this surface attraction is to compensate the attractive potential by introducing a repulsive optical potential using blue-detuned light [53, 59], thereby preventing atoms from reaching the surface. Another approach exploits the atom–surface interaction itself as part of the trapping potential to create stable traps near the dielectric surface [3, 60, 61]. The van der Waals contribution near the ONF can be calculated by modeling the fiber as an infinitely long silica cylinder [53], however, the dielectric nature of the nanofiber surface can be modified by atoms adsorbed onto the surface [62]. In addition to the Casimir-Polder interaction, charges accumulated on the nanofiber surface can generate a static electric field, which induces DC Stark shifts and gives rise to an additional attractive potential. Pennetta et al. [61] modeled this contribution as an electrostatic potential, $V_{\textrm{Q}}$, that depends on the DC polarizability of the atom and the linear charge density on the nanofiber surface. Owing to the distance dependence of the electric field produced by the charged nanofiber, this potential scales as $V_{\textrm{Q}} \propto -1/d^2$. Experimental observations validated a hybrid trapping model consisting of the optical trapping potential, the Casimir-Polder surface potential, and the electrostatic potential [61].

For trapping an atom near the ONF surface, an optical potential can be created by engineering the electric field close to the ONF surface. Such an electric field can be achieved using either evanescent trapping or a retro-reflection based (side-illumination) trapping scheme. The details of these two methods are described in the subsequent sections.

#### Evanescent traps

**Fig. 7:**
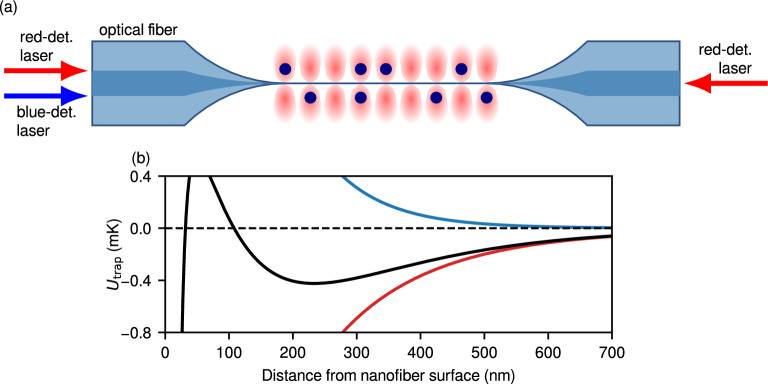
**a** Schematic diagram of atoms trapped near to the nanofiber surface in a two-color evanescent field trap formed by a guided blue-detuned laser and a pair of counter-propagating red-detuned lasers. **b** Trap potential extending radially from the nanofiber surface. Blue and red curves represent the potential due to the blue-detuned and red-detuned lasers respectively, with the black curve representing the overall potential, including surface forces from the nanofiber

One of the most common approaches to trapping atoms around a nanofiber employs a combination of two evanescent guided modes of opposite detuning with respect to the ground-state atomic transition. For a red-detuned evanescent field with a radially decaying intensity, an atom experiences an attractive force towards the nanofiber surface, while a similar blue-detuned field can serve as a repulsive force. As the evanescent decay length is shorter for the repulsive blue-detuned field due to having a shorter wavelength, an overall potential can be engineered such that it is repulsive near the nanofiber surface, and attractive further away. By additionally combining an axial standing-wave modulation using counter-propagating trapping fields, and by controlling the powers of each field, a stable potential minimum can be created 200–300 nm from the nanofiber surface, forming a one-dimensional array of trapping sites along the nanofiber axis. This is referred to as a two-color evanescent field dipole trap. The azimuthal confinement relies on the azimuthal asymmetry of the trapping field mode, and therefore requires precise control of the polarization. For quasi-linearly polarized $\textrm{HE}_{11}$ modes, two 1D arrays on opposite sides the nanofiber are created, as shown in Fig. 7. This configuration was theoretically proposed by Le Kien et al. [53], and first experimentally realized for cesium atoms by Vetsch et al. [59].

A significant complication arises from the locally elliptic polarization of the $\textrm{HE}_{11}$ mode in the evanescent field. This gives rise to a differential vector light shift which constitutes an important source of inhomogeneous broadening. The vector light shift from the forward-propagating guided mode can be canceled by a backward-propagating guided mode of the same wavelength, as the two contributions have opposite signs and largely cancel at the trapping site. Combined with the use of magic trapping wavelengths, where the differential scalar polarizabilities between the relevant states are suppressed, state-insensitive trap operation can be achieved [63, 64].

Trap lifetimes on the order of a few tens of milliseconds have been observed in such traps [59]; however, these lifetimes are far lower than that expected due to background gas collisions. A significant cause of heating can arise due to thermally occupied torsional modes of the nanofiber itself [65]. The torsional modes couple to the trapping field polarization and create a time-dependent perturbation of the trapping potential. The frequencies of these modes are found to be densely populated close to the trap frequencies, causing parametric heating of the atom in the trap.

In order to overcome the constraints on trap position and spacing due to the geometry of the original two-color trapping scheme, various proposals have been considered. A sub-*λ /2* spacing between trapping sites was realized using two red-detuned fields and a partial standing wave for the blue-detuned field [66], opening prospects for collective radiative effects such as selective radiance in atom arrays. Another recent proposal by Vylegzhanin et al. [67] combines a circularly-polarized free-space tweezer with the evanescent field of the nanofiber to trap rubidium atoms in a light-induced magnetic field trap. The beating of the two elliptical polarization components creates a spatially varying fictitious magnetic field that can trap ground-state $^{87}\textrm{Rb}$ atoms in a light-induced magnetic potential. In this configuration, the trap position can be continuously tuned over several hundred nanometers from the nanofiber surface by adjusting the relative optical powers. This allows *in situ* control of the atom coupling to the guided mode.

Atoms near nanofiber surfaces are also subject to non-optical surface forces—primarily the attractive Casimir–Polder interaction arising from quantum vacuum fluctuations, and electrostatic forces from surface charges accumulated on the fiber. In a recent work [61], a hybrid trap scheme was introduced, where surface forces replace the red-detuned field entirely.

#### Retro-reflection-based nano-traps

The two-color evanescent trap described in the previous section offers significantly improved control over the spatial localization of atoms near the ONF surface. However, this scheme provides little control over the actual number of atoms captured in the trap. Since loading generally occurs from a surrounding cold-atom cloud via stochastic processes, the occupancy fluctuates from shot to shot, yielding zero, one, or multiple atoms with no deterministic control. As a result, while the trap excels in positioning, it falls short of delivering the atom-number certainty required for deterministic quantum-network nodes [1]. In this context, a trapping technique based on retro-reflecting a tightly-focused external trapping beam from the nanostructure surface can be employed to achieve precise control over both the atom number and position. This allows the optical potential to be shaped independently of the guided modes, and allows atoms to be localized at predetermined sites with high spatial precision. Such trapping schemes have been employed to interface single atoms to nanostructures such as photonic crystal waveguides [68] and waveguide cavities [69–73], ONF cavities [74], and whispering-gallery-mode bottle micro-resonators [75].

The concept of this retro-reflection-based nano-trap (or side-illumination trap) was first demonstrated by Thompson et al. [69], where single atoms were interfaced with a photonic crystal waveguide. In this scheme, a tightly-focused laser beam directed onto a nanostructured surface forms a standing-wave interference pattern between the incident and reflected light, producing multiple trapping sites at positions of peak laser intensity. The characteristics of these retro-reflection-based traps, such as the distance from the surface to the first trap site and the effective trap depth, are determined by the wavelength of the trapping beam, and the dimensions and refractive index of the nanostructure [69]. Using this configuration, a nanophotonic quantum phase switch was demonstrated by trapping a single atom in the evanescent field of a nanoscale photonic crystal cavity [71]. A similar trapping scheme was employed to trap atoms in the near field of a photonic crystal waveguide, and super-radiance properties of atoms coupled to the waveguide were studied [68]. This technique has since been adapted to ONF systems [74], where real-time fluorescence of trapped atoms via the guided modes of the fiber was observed. Unlike evanescent-field-based traps for ONFs, where atoms enter the trap stochastically, the retro-reflection-based trap enables controlled loading protocols that can reliably capture a single atom, providing deterministic occupancy. The position of the nano-traps formed in relation to the surface of the nanostructure thereby achieve the positional stability required for implementing robust, cavity-enhanced light–matter interfaces. The approach enables atoms to be localized at predefined positions with high spatial precision.

**Fig. 8:**
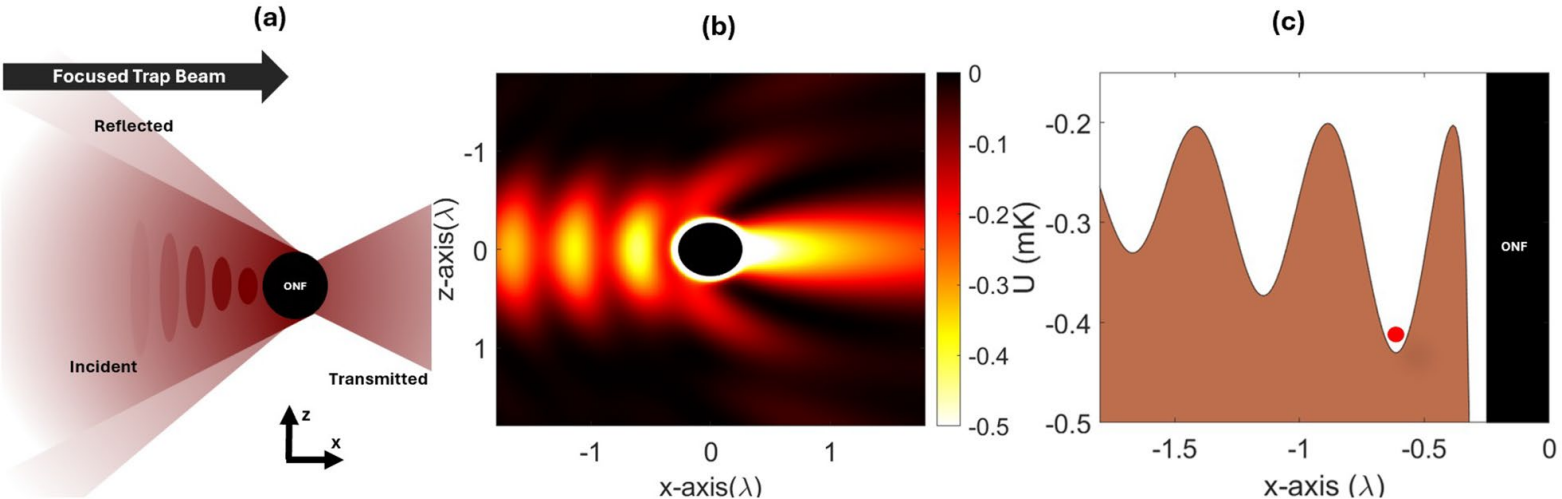
**a** Schematic of a retro-reflection based trap using an optical nanofiber (ONF). **b** 2D plot of the trapping potential experienced by a Cs atom. Beam waist, ONF diameter, trap beam wavelength and power are 1 *μ *m, 500 nm, 938 nm and 1 mW, respectively. **c** Trapping potential experienced by a Cs atom. Red circle indicates the deepest trap site

A schematic of a retro-reflection-based trap using an ONF, demonstrated by Nayak et al. [74] is shown in Fig. 8a. Here, the polarization of the incident beam plays an important role in deciding the final trap polarization. In the case of an incident beam with a polarization parallel to the nanofiber axis, the reflected light will have the same polarization and the resulting trap has a linear polarization. When the incident beam instead has a polarization perpendicular to the ONF, the reflected light will have a spatially varying polarization, which forms a trap with elliptical polarization. This introduces vector light shifts resulting in dephasing and a fluctuating dipole force, which can induce heating [69]. A more realistic model was simulated by considering a tightly-focused beam incident on a cylinder in Ref. [74]. The steady-state electromagnetic field distribution generated by the system was simulated using the finite-difference time-domain (FDTD) method, where the polarization was chosen to be aligned with the fiber axis. A typical potential experienced by a Cs atom is shown in Fig. 8b. The potential on the side of the ONF on which the beam is incident is shown in Fig. 8c, where the red circle indicates the deepest trap site. Although several interference maxima (potential minima) are generated, the evanescent field of the guided mode strongly overlaps with only the closest trapping site, leading to a single, well-defined trap near the ONF surface. As a result, the first trap site was used to estimate the characteristics of the nano-trap. The trap distance $d_\textrm{trap}$ is the distance between the surface of the ONF and the trap site, and determines the coupling efficiency of fluorescence into the guided mode of the ONF. The effective trap temperature $T_\textrm{trap}$ is defined as the difference between the potential minimum of the trap site, and the local potential maxima closest to the fiber, which is lowered due to the effect of the van der Waals potential of the ONF surface [53]. The diameter of the nanofiber determines the intensity and the phase of the reflected light, thereby deciding $d_\textrm{trap}$ and $T_\textrm{trap}$. The dependency of $d_\textrm{trap}$ and $T_\textrm{trap}$ was explored by simulating the system for various diameters, and the behavior was found to be periodic as reported in Ref. [74], which can be attributed to the phase shift experienced by the reflected light and its effect on the interference.

The coupling efficiency of an atom trapped in a nanofiber-based optical trap has been analyzed and quantified. The coupling efficiency *β * of a Cs atom to the guided mode of an ONF decreases with increasing atom–fiber separation. Studies indicate that *β * is significantly enhanced for ONF diameters in the range of 240–360 nm, however the trap depth $T_\textrm{trap}$ is lower than that for larger diameters (420–500 nm) [74]. This trade-off favors smaller diameters for applications that require strong light–matter interaction with a higher out-coupling rate. In particular, an ONF diameter that maximizes *β * is desirable to implement fast quantum gate cavity QED systems. With appropriate cavity integration, atoms interfaced to ONFs via retro-reflection-based trap schemes are promising for realizing nanofiber-based high-speed quantum gate operations for fault-tolerant quantum computing [76].

## Nanofiber and FBG cavity fabrication

### Fabrication of optimized optical nanofibers

The nanofibers described in Sect. 2.3 are typically fabricated using the heat and pull method [77]. Here, a piece of standard silica optical fiber is placed across two translation stages. A heat source at the center of the fiber softens the glass, and the fiber is stretched by moving the two translation stages apart, forming a tapered fiber. Since typical heat sources are quite large and usually do not have a uniform heating profile, the flame brush technique [77, 78] is employed to mimic a uniform heat source of length *L*. In this technique, a small heat source is moved back and forth over *L*, with a constant speed much faster than that of the pulling stages. This allows for experimental taper profiles much closer to theoretical profiles, giving greater control over their fabrication. Varying this hot-zone over the course of the pulling can also allow for, e.g., ONFs with long uniform waists to be produced [77].

#### Heat sources for optical nanofiber fabrication

The most common heat source used for producing nanofibers is a small gas flame [49, 77–97], often using oxy-hydrogen [86, 87, 90–93, 96, 97], pure hydrogen [49, 80, 94], oxygen-isobutane [83], or butane [85] gas, with the flame typically of millimeter size. This method is capable of producing nanofibers with sub-micron diameters and high transmission [49, 92, 95, 97], so long as the gas flow line is clean, the gas supply is of high purity, and the flame shape is precisely controlled [83]. Hydrogen and oxy-hydrogen flames are typically cleaner as they only produce water as a byproduct [92], and they also produce higher temperatures at lower gas rates compared to butane, which is beneficial for reducing pressure exerted on the fiber that can cause slight deformations in the nanofiber profile [95].

Taper pullers utilizing a CO_2_ laser to heat the fiber have also been reported [98–103]. This method has some advantages over flame-based tapering, as there is no added force on the fiber, and no contaminants produced [99, 100]. The heating process is extremely fast, and the laser spot can be made much smaller than a flame, closer to the point-source assumed in modeling of tapers, allowing for greater control of the taper profile [100]. The technique can be further improved by heating the fiber with the beam from both sides to produce more uniform tapers with low ellipticity [102]. Using a CO_2_ laser also makes the tapering process self-regulating; when a laser beam of fixed power is used, the fiber will eventually reach a small enough diameter that it cannot absorb sufficient energy from the laser to reach the softening point [99, 104]. On the other hand, extremely high power is required in order to produce sub-micron fibers by direct heating with a CO_2_ laser. Additionally, power fluctuations of the laser can cause fluctuations in the fiber diameter [100], and numerical modeling shows oscillations in the fiber temperature that are dependent on fiber diameter and laser polarization [104], which may further limit CO_2_-laser-based taper pullers for ONF fabrication.

Electric microheaters have also been used as a heat source [105–110]. While such heaters can produce sub-micron fibers, they are generally large in size, so the tapers made are typically very long, and heat transfer is slow [100]. The temperature profile of the heater is generally not constant along the length, though it is possible to account for this through calibration of the tapering properties [110]. There are also concerns that contaminants ejected from ceramic microheaters can cause extra losses in the final taper [99].

A related method involves heating a small ceramic or sapphire tube to make a microfurnace [111–113]. Here, the optical fiber is placed within the tube that is then heated externally, usually by CO_2_ laser. The size of the hot-zone can be controlled by adjusting the focus of the laser beam. Sub-micron fibers are able to be produced [111, 112], with much shorter lengths than those made using electric microheaters. This method provides a very clean environment for tapering, and reduces air flow around the fiber that may cause deformations in the taper profile [112]. Similarly, a sapphire tip heated by a gas flame has been used to make nanofibers in a secondary pull step [82], and to produce nanowires [114].

Aside from using the heat and pull method, fiber tapers can also be produced by chemical etching of the fiber [115–119]. However, the etching process generally produces tapers with comparatively poor surface roughness, leading to high losses.

#### Producing ultra-low-loss nanofibers

For use in cavity QED experiments, nanofibers with low transmission losses are desired. A number of factors contribute to the success of tapering low-loss nanofibers. Surface contaminants can cause loss, so the fiber must be thoroughly cleaned before tapering, and the taper pulling should be undertaken in a clean environment [91, 92, 109]. The heat source used will also contribute to the cleanliness as discussed above. Surface roughness also needs to be minimized to ensure low loss.

The power coupling from the fundamental mode into higher-order modes due to the changing radius of the fiber in the taper region can also cause significant losses. Such losses can be minimized by ensuring the taper profile is adiabatic [49, 50, 77, 120]. A number of studies have shown adiabatic nanofibers with transmission above 99% [87, 91, 92], however the small local taper angle produces nanofibers with a long overall length. It is also possible to manufacture shorter nanofibers while still maintaining a high transmission by varying the local taper angle such that it remains below some fraction of the delineation angle defining the mode coupling in the fiber [49, 50, 77, 120] (see Eq. 42 in Sect. 2.3). Such short high transmission nanofibers are useful when high mechanical stability is required, or in experiments with limited space.

Another factor that limits transmission in silica nanofibers is water absorption. This is an issue particularly in experiments with ytterbium atoms, where the operation wavelength is close to the water peak in silica fibers. It has been shown that these absorption losses can be suppressed by replacing the oxy-hydrogen flame with a deuterium-oxygen flame [121].

The presence of water and other air molecules when heating the fiber can also lead to devitrification [109, 122, 123], where molecules such as water produce nucleation sites for crystal formation on the fiber at high temperatures. For standard fiber, the transmission is generally unaffected by crystals on the fiber surface, but on a nanofiber they can produce large losses. The process can occur quickly at normal tapering temperatures [122], though it can be somewhat reduced by rapidly heating and cooling the fiber. Alternatively, an argon environment can be used to suppress devitrification, though this is incompatible with a gas flame heat source. Additionally, if the fiber is heated to temperatures above 1500 °C, *β *-cristobalite formation can occur, and these crystals remain if the fiber is rapidly cooled, leading to losses [93].

Finally, nanofibers below a certain diameter exhibit a strong decrease in transmission. As the propagation constant of the fundamental guided mode reaches that of the radiation mode, the fundamental mode experiences substantial loss until it completely disappears [124, 125]. In fact, experimentally produced nanofibers exhibit even stronger losses, possibly from small non-uniformities arising during the tapering process.

#### Nanofiber optimization for QED experiments

In order to ensure the best performance of QED experiments with nanofibers, the radius of the nanofiber waist and the transmission need to be optimized to obtain high cooperativity when interacting with atoms. The specific size will depend on the particular atom species used and the trapping method. Due to the small size of a nanofiber, they cannot be measured accurately with an optical microscope. Although a relatively accurate measurement of the waist size can be obtained with a scanning electron microscope, the process is destructive and the nanofiber cannot be used again afterwards [126, 127]. Measuring the fiber *in situ* without damaging it is ideal.

The size of a tapered fiber can be measured by observing the scattering pattern of light illuminating the fiber from the side [126]. The nanofiber size able to be measured is limited by the illumination source wavelength, though the accuracy is low, around *± 50* nm.

Second- and third-harmonic generation has also been used to measure the size of nanofibers through intermodal phase-matching [90, 113, 127]. Here, efficient harmonic generation occurs when the modes of the fundamental and harmonic light are phase-matched. Since the phase-matching conditions will be determined by the exact diameter of the fiber, the diameter can be found from the wavelength of the generated harmonic light. However, it is possible for the fiber to be damaged using this method if UV light is produced in the harmonic generation process [127].

Another method involves bringing a nanofiber into contact with an external nanostructured cavity containing a defect, creating a cavity for the fundamental nanofiber-guided mode [128]. The sensitivity of the effective refractive index to the nanofiber diameter causes the cavity resonant wavelength to shift depending on the placement of the external nanostructure, and allows for *in situ* nanofiber diameter measurements, with a precision up to 10 nm.

A method that allows the nanofiber radius to be monitored during the pulling process is the observation of the spectrogram of a transmitted laser [91, 95]. From the spectrograms, the mode cutoffs for each higher mode can be observed over the course of the taper pulling. Since the mode cutoffs of a particular wavelength laser will occur at certain fiber radii, it is possible to determine the radius of the nanofiber in real time, so the pulling process can be stopped when the fiber has reached the desired radius. The mode cutoffs can also be observed by a drop in power in the laser transmission, without calculating the spectrogram [94].

To monitor the nanofiber loss, the transmission of the required wavelength can be simply measured during the tapering process [90, 92, 103], though it is difficult to precisely measure ultra-low-loss nanofibers in this way due to fluctuations of the laser intensity or polarization. A more accurate measurement can be obtained by pulling a nanofiber in the middle of an optical cavity composed of two fiber Bragg gratings [95]. Instead of measuring transmission, the finesse of the cavity can be measured over the course of the tapering by a ringdown measurement [129], which is independent of laser fluctuations.

### Nanofiber fiber Bragg grating cavities

Integrating cavity structures into an ONF is a key requirement for establishing optical nanofibers as a platform for cavity QED [59]. A cavity containing an ONF can be realized by fabricating mirrors surrounding the subwavelength waist section of the fiber. To this end, two main approaches have been developed. Firstly, mirrors can be fabricated outside of the taper region in the standard diameter optical fiber. Secondly, the mirrors can be fabricated directly on the nanofiber waist, thereby avoiding taper-related losses [55]. The former approach is reviewed in this section, while the latter is discussed in Sect. 3.3.

#### Fiber Bragg grating cavities: basics, fabrication and applications

Mirrors on standard optical fiber are typically realized using fiber Bragg gratings (FBGs), the fabrication of which is a well-established technique and has been widely used in the telecommunications industry [130–132]. Fiber Bragg grating mirrors are created by introducing a controlled refractive-index modulation in the fiber core through modification of the material properties. These mirrors are typically fabricated using deep-ultraviolet (DUV) photo-inscription through a phase-mask technique, which enables a precise and reproducible periodic index modulation in the fiber core [130, 133]. This process relies on the intrinsic photosensitivity of Ge-doped silica fibers, first observed by Hill et al. [134], and later attributed to UV-induced structural modifications in the glass matrix [135]. The photosensitivity can be significantly enhanced by hydrogen loading, which increases the efficiency of refractive index modification under UV exposure [136, 137]. Because this is a non-contact, non-ablative technique, it avoids surface damage, contamination, and material removal, enabling the fabrication of mirrors with exceptionally low optical loss and high reproducibility. The optical properties of UV-induced FBGs have been investigated and formulated by Erdogan [138].

The refractive index modulation of an FBG forms a narrow stop-band in transmission, with the resonance at the Bragg wavelength $\lambda _\textrm{B}$, given by

54 $$\begin{aligned} \lambda _\textrm{B} = 2 n_\textrm{eff} \Lambda _\textrm{G}, \end{aligned}$$

where $n_\textrm{eff}$ and $\Lambda _\textrm{G}$ are the effective refractive index and period of the refractive index modulation, respectively. The resonance defining parameters, $n_\textrm{eff}$ and $\Lambda _\textrm{G}$, are strongly modified by external perturbations such as strain and temperature, leading to a measurable change in resonance wavelength. The simplicity, robustness, and multiplexing capability of FBGs allow them to be widely employed in sensing applications, particularly in strain and temperature measurements, with practical applications such as structural monitoring and vibration sensors [139, 140]. Fiber-based optical filters and optical signal processing units have also been implemented using FBGs by engineering the refractive index modulation profile [141].

It has been demonstrated that by using two FBGs to form a fiber-based Fabry–Pérot cavity, narrow resonances can be achieved, enabling high spectral resolution [142]. As a result, FBG cavities have been extensively explored for sensing applications. Small perturbations in strain, temperature, or refractive index can be efficiently converted into sharp resonance shifts, enabling significantly enhanced sensitivity compared to single-FBG sensors [139, 143]. Such cavity-enhanced FBG sensors have been widely applied in structural monitoring, environmental sensing, and biochemical detection [144].

**Fig. 9:**
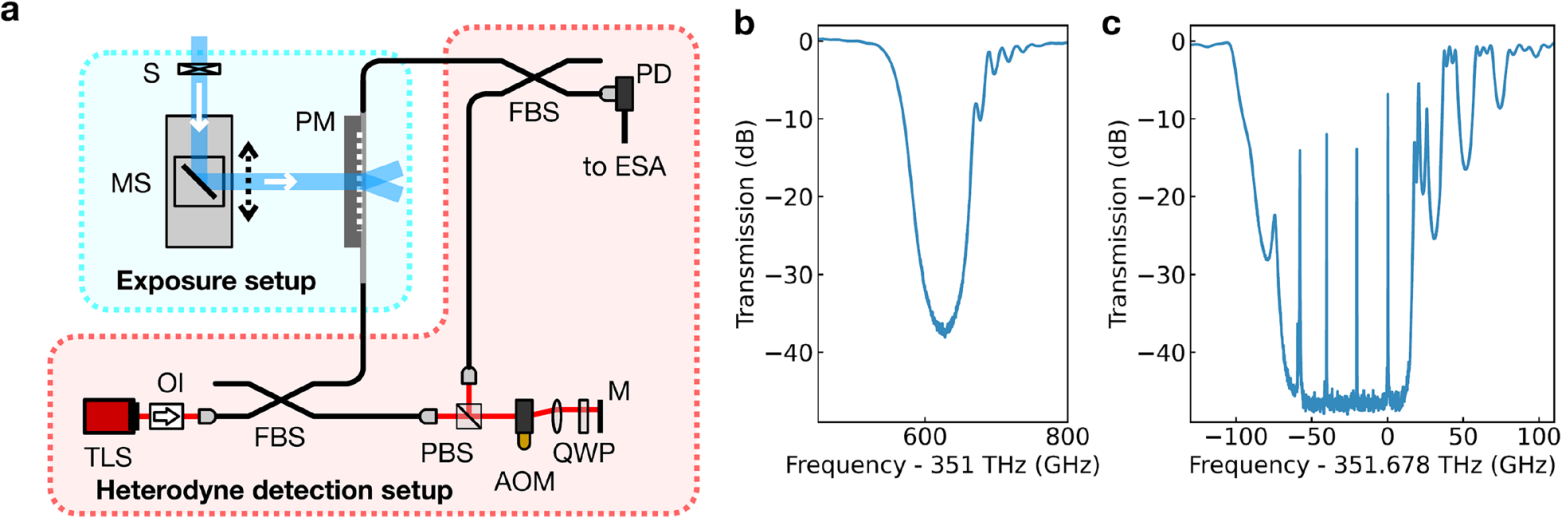
**a** Schematic of the FBG fabrication and characterization set-up. S: Mechanical Shutter, MS: Motorized stage, PM: Phase Mask, TLS: Tunable laser source, OI: Optical Isolator, FBS: Fiber beam splitter, AOM: Acousto-optic modulator, QWP: quarter-wave plate, M: mirror, PD: photodetector, ESA: Electro spectrum analyzer. **b** Transmission spectrum of a typical single FBG. **c** Transmission spectrum of a FBG cavity. Reprinted with permission from Ref. [145]. © Optica Publishing Group

A schematic diagram of the fabrication and characterization of the FBG cavities used in Ref. [145] is shown in Fig. 9a (cyan shaded region). A DUV laser is passed though a phasemask designed according to the target wavelength, diffracting the beam into ±1 orders which combine to form an interference pattern close to the surface of the phasemask. A stripped single-mode silica fiber (SM800, Fibercore) is placed within the interference pattern, where the photo-sensitivity of the fiber core allows for the creation of a periodic refractive index change to form an FBG. The Q-switched DUV laser has a wavelength, power, and pulse repetition rate of 213 nm, 100 mW and 15 kHz, respectively [95, 145]. The DUV laser beam can be scanned along the fiber, extending the FBG length, and thus increasing the reflectivity. Since the inscription is carried out on standard optical fiber rather than a nanofiber section, the technique can be quite robust against mechanical vibrations. The transmission spectra of the FBGs are measured using the heterodyne detection setup shown in Fig. 9a (red shaded region), and examples of a single FBG and an FBG cavity transmission spectra are shown in Fig. 9b and c respectively. The observed stop-band is narrow, with a bandwidth of only a few tens of GHz.

The resonance wavelengths of the FBGs are determined by the properties and parameters of the fiber, phase mask and the DUV laser wavelength. However, due to the narrow stop-band, tuning and stabilization are required to correct against fabrication imperfections. Temperature and strain tuning can be used to match the FBG resonance, as well as the FBG cavity modes, to a target atomic transition [47, 146]. Additionally, the FBG resonance can be tuned towards shorter wavelengths by pre-straining the fiber prior to fabrication. An FBG-nanofiber cavity was used to study the optically-active mechanical modes, which modulate the phase and polarization of the guided light via the strain-optic-effect [65]. Thermalization behavior of a nanofiber thinner than the thermal wavelength was investigated by using an FBG cavity to measure the thermo-optic effect via cavity mode resonance shifts [147]. Kato et al. fabricated FBG cavities with finesse of 8600 ($Q = 1.5 \times 10^8$) and demonstrated a single-frequency fiber Fabry–Pérot Brillouin laser [145].

#### Nanofiber FBG cavities for cavity QED

Liang et al. [148] fabricated a tapered fiber between two FBGs to form a cavity, and coupled light into a micro-toroidal resonator, observing a variety of resonance spectral features of the coupled system. Inspired by this concept, Wuttke et al. [149] demonstrated an all-fiber Fabry–Pérot cavity, with the nanofiber waist serving as the interaction region for atoms coupled to the evanescent field of the guided mode, and showed that this micro-resonator is compatible with the observation of coherent cavity QED, such as Rabi oscillations [47]. Such systems can be used to realize vacuum compatible, fully fiber-integrated cavities without damaging the nanofiber waist. This architecture has enabled strong atom–cavity coupling in a fully fiber-integrated platform [47], building on earlier demonstrations of nanofiber-based atom trapping [59, 64].

In the context of an all-fiber quantum network, a system with two FBG nanofiber cavities has been studied [8, 150–152]. Normal modes of this coupled system were investigated both theoretically and experimentally, and shown that by using the “cavity dark mode”, remote excitation and non-local saturation can be achieved without photon excitation at the locations of the atoms [151], making it a promising candidate for realizing a quantum network. However, practical implementation requires higher coupling efficiency to quantum channels. In this context, Ruddell et al. [95] demonstrated ultra-low loss FBG-nanofiber cavities optimized for cavity QED, and achieved a finesse of 1480, corresponding to a cooperativity $C_{in}>500$ for a Cs atom trapped 200 nm away from the surface. By including the total round-trip loss of only 0.34% in the model, $C_{in}$ is decreased to 150. Horikawa et al. [97] optimized the fabrication process and achieved a finesse of 3600, corresponding to a round-trip loss * = 0.14(1)*%. Methods to characterize and store such high-finesse FBG nanofiber cavities in a portable container were also developed, enabling on-demand installation into atom experiments. The prospects of such FBG-nanofiber cavities for implementing fault-tolerant quantum computing (FTQC) is discussed in Ref. [76].

### Nanofiber photonic crystal resonators

Integrating a cavity directly into the nanofiber section in the form of a photonic crystal (PhC) can potentially offer several advantages compared to alternative types of nanofiber cavity. In particular, the ability to design extremely short cavities can more easily provide access to the Purcell regime, where $\kappa \gg g \gg \gamma$ [2]. Additionally, by having the tapered sections of fiber outside of the cavity, the loss associated with those sections can be excluded from the cavity system. Nanofiber PhC cavities are typically fabricated as a physical structure embedded into the nanofiber using either focused ion beam (FIB) milling techniques [153, 154] or femtosecond laser-induced ablation [155, 156]. Composite nanofiber cavities combining a nanofiber with an external PhC cavity have also been demonstrated [157].

#### Focused ion beam milling

By accelerating an ion beam and allowing it to collide with a target material, such as an optical fiber, atoms can be ejected from the material by a process known as sputtering. Through careful control of parameters and by steering of the ion beam, it is then possible to mill patterns into the material surface. The high level of control allows patterns to be created essentially arbitrarily, and therefore FIB milling presents a promising method for fabricating cavity structures on an optical nanofiber. Nevertheless, the highly curved surface and large aspect-ratio of the nanofiber creates difficulties for milling due to both mechanical vibrations and charge accumulation on the silica fiber causing misalignment. Additionally, as atoms are ejected from the material during the fabrication process, care must be taken that they are not redeposited onto the nanofiber surface. An extensive review regarding the FIB milling of optical nanofibers has been previously presented by Romagnoli et al. [153], and hence we provide an overview of various implementations here.

Nayak et al. [158] demonstrated the fabrication of nanofiber PhC cavities using FIB milling with Ga$^+$ ions, targeting an *∼ 800*–850 nm resonant wavelength. Here, a grooved PhC was formed on a nanofiber with diameter of *∼ 560* nm, with each groove having a depth of *∼ 100* nm and a width of *∼ 150* nm, and an overall grating period of *∼ 360* nm. A cavity was then fabricated from two PhCs each having 120 grating periods, separated by a *∼ 100* um bare nanofiber defect. The physical structure of the PhC breaks the cylindrical symmetry of the system, and hence the effective refractive index differed between the *x*- and *y*-polarizations, resulting in differing cavity transmission properties for each polarization mode. The highest quality mode was observed to have a *Q*-factor of $\sim 5\times 10^4$ with a free spectral range (FSR) of *∼ 810* GHz.

Ding et al. [159] demonstrated FIB milling of a microfiber cavity structure on a 2.3 *μ *m microfiber, achieving a *Q*-factor of *∼ 60* at a wavelength of 1180 nm.

Li et al. designed and fabricated compact cavities using a gallium FIB process by milling square grooves having a width of *∼ 100* nm and period of *∼ 310* nm in an *∼ 830* nm diameter fiber. They formed a cavity with a defect distance of *2~μ *m and a resonant wavelength of *∼ 780* nm, achieving an estimated *Q*-factor of *784± 87* for a 20-period grating [160].

Schell et al. [161–165] fabricated a PhC cavity targeting a 630 nm resonance wavelength to efficiently couple photon emission from colloidal quantum dots. Their cavity was formed using a nanofiber diameter of 270 nm, a groove depth of 45 nm, spacing of 300 nm, with a total of 160 periods and with a 450 nm center defect. The resulting *Q*-factor of *∼ 250* was possibly limited by drifts during their FIB fabrication process [161]. Takashima et al. [163] then improved their gallium FIB fabrication process to achieve a *Q*-factor of 450. To overcome the contamination associated with deposition of gallium ions during fabrication, and for improved accuracy down to 1 nm, they also used a focused helium ion beam process. Here, cavities were fabricated with up to 640 periods total on a nanofiber diameter of *∼ 310* nm, with a groove depth of 30 nm, pitch of 320 nm, and a defect of 840 nm between the two gratings. In this way they were able to fabricate a device having a resonant wavelength of 699.80 nm and a *Q*-factor exceeding 4170. Tashima et al. [164] used a helium FIB to fabricate a cavity on a nanofiber with a diameter of *∼ 400* nm, consisting of two PhCs separated by a 755 nm defect, each having 250 periods with a groove depth of *∼ 10* nm and groove spacing of *∼ 200* nm. They achieved a *Q*-factor of 807 at a resonant wavelength of *∼ 573* nm to match the fluorescence wavelength of a hexagonal boron nitride nanoflake. Takashima et al. [165] then numerically studied the mechanism of ultrawide tunability in nanofiber PhC cavities under mechanical stress, and found that both the elongation of the grating period as well as a slight shrinking of the nanofiber diameter contribute to the tuning.

To prevent charge accumulation during fabrication, Wuttke [166] fixed a nanofiber to a polished copper substrate by Van der Waals forces to allow the nanofiber to discharge during the process, and was able to fabricate PhC cavities having a maximum *Q*-factor of $9.8\times 10^4$ at a resonant wavelength of 838 nm. Wuttke noted that performance was limited due to contamination by both pollutants and gallium ions during the fabrication process, and showed that implementing an annealing procedure could recover some transmission post-fabrication.

FIB milling techniques also allow the opportunity to enhance waveguides in other ways, such as creating a nanogroove slot in the center of the waveguide to house an emitter and enhance the photon emission and coupling efficiency [167–170].

The ability to create arbitrary structures makes FIB milling a promising tool for fabricating highly tailored nano-photonic devices [153, 154]. Nevertheless, the technique can be time consuming, and requires mounting the nanofiber within special equipment. Additional challenges include avoiding contamination from the ion beam and substrate, as well as avoiding mechanical instability caused by charging of the nanofiber during the fabrication process. Alternative optical-based methods have also been explored for fabricating nanofiber PhC cavities.

#### Femtosecond laser ablation

**Fig. 10:**
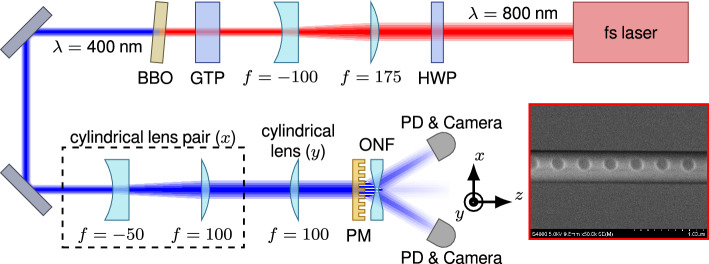
Experimental setup for fabricating PhC cavities on nanofibers by femtosecond laser ablation. HWP: half-wave plate, GTP: Glan-Taylor polarizer, BBO: barium borate crystal, PM: phase mask, ONF: optical nanofiber, PD: photodiode. Lens focal length units are given in mm. Inset: SEM image of a section of a PhC cavity fabricated using this setup. Reprinted with permission from [156]. *\copyright * Optica Publishing Group

By exposing a nanofiber to a periodic intensity pattern from a single high-power femtosecond laser pulse, it has been shown that a PhC consisting of a periodic array of craters can be realized when material is ablated at the positions of peak intensity. An example of an exposure system for optical fabrication of PhC nanofiber cavities is shown in Fig. 10, utilizing a femtosecond laser frequency doubled to 400 nm [156]. To maximize the intensity of the laser light on the nanofiber, the beam is expanded along the nanofiber axis using a pair of cylindrical lenses, before being focused to a line using a cylindrical lens in the orthogonal direction. The beam passes through a phase mask, designed to diffract light into the *± 1* orders, which interfere to create a periodic intensity pattern immediately behind the phase mask, where the nanofiber is positioned, similar to the FBG fabrication method presented in Sect. 3.2.1 for standard fiber. The light hits the nanofiber, with the front face of the nanofiber acting as a cylindrical lens and further focusing the light onto the rear of the nanofiber, where material is ablated. Here, periodic craters can be formed using a single femtosecond pulse, with the size of the craters following the Gaussian profile of the femtosecond laser beam intensity distribution. A SEM image showing a section of a nanofiber PhC cavity can be seen in the inset of Fig. 10. The variation of crater size with laser intensity causes an apodized effective refractive index variation along the PhC, which allows for the creation of nanofiber PhC cavities where it is not necessary to introduce a defect region between two PhCs to form the cavity.

Nayak et al. [155, 171–174] demonstrated the fabrication of PhC nanofiber cavities using femtosecond laser ablation for nanofibers with diameters of around 450–500 nm. Cavities were fabricated with and without a central defect, and a maximum finesse of 500 at a wavelength of *∼ 795* nm was observed, corresponding to a *Q*-factor of $1.6\times 10^6$. SEM measurements of the cavity structure revealed circular craters with diameters of 50–250 nm varying with the laser intensity profile, and spaced by 350 nm over a length of 1 mm [155]. Keloth et al. [172] demonstrated the fabrication of a centimeter-scale PhC cavity directly on a nanofiber. By creating a taper with a 1.7 cm long waist, and having a diameter of *500± 2* nm over the entire waist length, they were able to fabricate two PhC mirrors separated by 1.2 cm using femtosecond laser ablation. The resulting cavity was shown to be suitable for experiments in both the Purcell and strong-coupling regimes of cavity QED, with an estimated cooperativity *C* ranging from 3 to 25 for measured cavity modes. Wang et al. [174] investigated photothermal properties and thermal self-locking in a centimeter-scale nanofiber PhC cavity with an effective length of *∼ 18.2* mm. It was found that the cavity modes could be tuned at a rate of 250 GHz/mW (*∼ 0.5* nm/mW) by using off-resonant light at 830 nm. By using light resonant with a cavity mode, thermal self-locking was also demonstrated using just a few *μ *W of light.

Tanaka et al. [156] have demonstrated defect-free nanofiber PhC cavities having *Q*-factors exceeding $10^7$, more than one order of magnitude higher than previously reported [155]. An investigation into the nonlinear properties of these cavities revealed a large thermal response, greatly reducing the threshold for optical bistability compared to that for the pure Kerr effect. Additionally, the nonlinear thermal response remained dominant across the entire cavity bandwidth, even when interrogating with pulses more than one order of magnitude shorter than the 6.6 *μ *s thermal cutoff time.

#### Composite PhC cavities

It has been shown that composite PhC devices can be formed by combining a bare nanofiber with an external periodic grating. Here, the external grating causes a periodic modulation of the cladding-mode refractive index, allowing for the realization of a Bragg reflector for nanofiber guided modes. Sadgrove et al. [128, 157, 175] have demonstrated Bragg reflection by such a device by bringing a nanofiber into contact with an external grating, and observed a broad reflection spectrum with a bandwidth up to 9 nm near a center wavelength of 765 nm [157]. This method was extended to create a cavity for the nanofiber guided mode by utilizing an external grating containing a central defect [175]. A nanofiber with a diameter of 550–600 nm was mounted on a grating with 350 periods, with a grating spacing of 320 nm, and a central defect of 480 nm. The asymmetry of the refractive index modulation lifts the degeneracy of orthogonally polarized modes, with *Q*-factors of *1270± 20* and *2310± 80* measured experimentally. Simulations suggested an effective cavity length of 28 *μ *m, with an estimated finesse of *28± 1*. Keloth et al. [128] have also demonstrated nanofiber diameter measurements using an external defect grating, as discussed in Sect. 3.1.3. By bringing a nanofiber into contact with an external nanostructured cavity containing a defect, *in situ* nanofiber diameter measurements were realized, with an achieved precision as high as 10 nm. Yalla et al. [176–178] have demonstrated a tunable composite cavity by utilizing a symmetrical external defect-mode grating designed such that the grating period diverged linearly, so that the grating period experienced by the nanofiber guided mode could be adjusted by moving the grating perpendicularly to the nanofiber. The resulting cavity could be tuned by ±10 nm around a center wavelength of 640 nm [176]. By further allowing the number of grating periods on each side of the grating defect to be asymmetrical, a one-sided tunable composite cavity was also demonstrated [177, 178].

## Nanofiber QED experiments

### Review of nanofiber-atom experiments

Initial cold-atom experiments with ONFs demonstrated that the atomic energy levels and the decay properties are strongly modified around the ONF. Nayak et al. [54] demonstrated that atomic fluorescence from even a few cesium atoms surrounding the ONF could be efficiently collected into a guided mode and detected at the fiber ends. The fluorescence spectrum showed the characteristic asymmetric red-side broadening attributed to the van der Waals interaction, an unavoidable surface effect in such systems.

At the same time, Sague et al. [179] performed in-fiber absorption-spectroscopy of a cold cesium cloud by investigating the transmission of the low-power laser probe through the fiber. The transmission spectrum showed a lineshape that was asymmetrically broadened and red-shifted from the free-space resonance. At resonance, as few as two atoms (on average) in the evanescent-field absorbed 20% of the total power transmitted through the fiber. Critically, they measured a broadened absorption spectrum even at vanishingly low powers, in agreement with significant enhancement of the spontaneous emission rate near the nanofiber surface.

Nayak et al. [180] extended the fluorescence technique to detect individual cesium atoms that transit the evanescent field. They measured $g^{(2)}(\tau )$ correlation of the fiber-coupled fluorescence photons and observed a clear dip at *τ = 0* below the classical limit, thereby observing anti-bunching and confirming single atom detection. For a single two-level atom, $g^{(2)}(0) = 0$ (photon anti-bunching), since an atom cannot emit two photons simultaneously. The same group later investigated how the photon correlation statistics in resonance fluorescence evolve as the number of atoms changes [181]. They showed that, when the photons are detected at the same fiber end (same propagation direction), the correlation varies from anti-bunching to bunching as the atom number increases, reflecting the transition from a single-emitter to a multi-emitter source. When photons from the two ends of the fiber are correlated (opposite propagation directions), the correlations remain anti-bunched regardless of atom number, a consequence of the spatial mode structure of the guided field. These results characterized the statistical nature of the coupled fluorescence and established the ONF platform as a tool for few-atom quantum optics.

These initial studies, centered on fluorescence coupling, laid the groundwork for a different class of experiments. Several groups used an ONF as a sensitive probe to measure macroscopic properties of a cold atom cloud. Morrissey et al. [182] used an ONF embedded in a MOT to characterize its cloud shape and size and to measure the atom number and loading rate. Russell et al. [183] measured sub-Doppler temperature and its variations on the order of *μ *K by comparing the ONF-collected coupled fluorescence signal through different stages of a release-and-recapture process, and used it to diagnose the misalignments in their MOT setup. In addition, while dithering the magnetic trapping field, they could extract the trap frequency and corroborated it with standard free-space measurements. Later, Grover et al. [184] extracted atomic-cloud temperature via photon-correlation measurements of the coupled fluorescence. The temporal width of the intensity-correlation corresponds to the atom transit time through the evanescent field and thus the most probable atomic velocity, hence the temperature. They confirmed the results with standard time-of-flight measurements and atomic trajectory simulations.

As noted before, a very low probe laser power (sub-nW) is sufficient to saturate atoms, and as such ONFs are highly efficient tools for nonlinear optics. Hendrickson et al. [185] demonstrated two-photon absorption in rubidium vapor surrounding an ONF. Rajasree et al. [186] later demonstrated a degenerate two-photon transition, which is a much weaker nonlinear process and otherwise requires very high intensities. Pittman et al. [187] measured nonlinear transmission and low-power saturation through a nanofiber surrounded by metastable xenon atoms. Spillane et al. [188] demonstrated nonlinear absorption and V-type electromagnetically induced transparency (EIT) in Rb vapor. Two other experiments [189, 190] realized EIT in ladder configuration. Kumar et al. [190] observed Autler-Townes splitting and frequency up-conversion at probe powers of less than 20 nW. Furthermore, a similar ladder-type excitation was used to excite atoms to Rydberg state [191, 192] near an ONF surface. Integration of Rydberg atoms with ONFs may open paths to quantum networking via optical photons.

The decisive advance that transformed the ONF from a spectroscopy platform into a quantum interface was the realization of a stable atom trap around the ONF by Vetsch et al. [59]. Using a red-detuned beam at 1064 nm and a blue-detuned beam at 780 nm guided through a 500 nm diameter nanofiber, they trapped an ensemble of  2000 Cs atoms, with an optical depth of 32, corresponding to 1.6% average absorbance per atom—a hallmark figure of merit. Lee et al. [193] adapted the trapping scheme for Rb atoms, albeit reporting trapping of only 300 atoms. This was attributed to significantly larger differential shift in the case of Rb atoms. Gupta et al. [194] investigated machine learning approach to improve the loading of Rb atoms in such a trap, and achieved a 50% increase in the number of trapped atoms. More recently, Pache et al. [66] realized sub-*λ /2* spacing between trapping sites using two red-detuned fields and partial standing wave for the blue-detuned field, opening prospects for collective radiative effects such as selective radiance in atom arrays.

Dawkins et al. [195] performed dispersive interaction with trapped Cs atoms. In the dispersive regime, the atoms impart a phase shift on the transmitted light with minimal absorption, enabling non-destructive atom number measurement from a single probe pulse. The measured per-atom phase shift was approximately 1 mrad at a detuning of *6Γ *. Beguin et al. [196] used a similar technique and measured the differential phase-shift between equally detuned red- and blue-detuned probes via homodyne interferometry to extract atom number distribution per site in their ONF-coupled Rb array, and found it to be sub-Poissonian. This sub-Poissonian loading arises from collisional blockade: the strong short-range atom–atom interaction suppresses double occupancy of a single trapping site.

A general concern with two-color trapping is inhomogeneous dephasing due to differential light shift between hyperfine states. Goban et al. [64] addressed this directly by demonstrating a state-insensitive, compensated trap using magic wavelength trapping fields in a standing-wave configuration, achieving near-natural-linewidth spectroscopy of trapped Cs atoms. While in general efforts are made to suppress the differential shift, Albrecht et al. [197] demonstrated its application for probing and manipulation of the motional state of atoms. By tuning the fictitious field strength via an external magnetic field or a tune-out wavelength laser, they controlled the sideband coupling and implemented microwave sideband cooling to a mean motional quantum number of $\langle n\rangle = 0.3$, near the motional ground state in one dimension. Meng et al. [198] later demonstrated near-ground-state cooling using Raman sideband cooling along the radial direction, reaching motional occupation $\bar{n} < 1,$ a significant step toward full quantum control of the motional state.

Reitz et al. [199] conducted the first systematic study of coherence properties in such a trap. Using microwave pulses to drive the Cs clock transition, they recorded Ramsey fringes and spin-echo signals. They measured a reversible dephasing time $T^*_2 = 0.6~\text {ms}$ and an irreversible dephasing time of $T_2^{\prime } = 3.7~\text {ms}$. Both timescales are limited primarily by the finite initial temperature of the atomic ensemble and the heating rate of the trapped atoms. These measurements set concrete benchmarks for subsequent improvements. Pennetta et al. [61] demonstrated experimentally a hybrid trap scheme where surface forces replace the red-detuned field entirely. In this hybrid scheme, cesium atoms loaded from the conventional two-color trap are adiabatically transferred into a trap where the stable confinement is provided only with the blue-detuned repulsive field and the surface attraction. Despite its shallow depth ($\sim 1~\mathrm {\mu }K$), the hybrid trap yields a storage time of $140(9)~\textrm{ms}$ and a coherence time ($T^*_2$) of $16.8(2)~\textrm{ms}$—the latter exceeding significantly all previous nanophotonic benchmarks.

When multiple atoms are coupled to the same 1D guided mode, they interact through the exchange of guided photons over arbitrarily long distance—a form of infinite-range dipole–dipole interaction unique to 1D waveguides. This coupled with the high optical depth of ONF-trapped atom arrays opens the door to nanofiber-based waveguide quantum electrodynamics.

The ONF experiments with laser-cooled atoms discussed above have predominantly employed alkali species such as Cs and Rb, whereas recent work has begun to extend this platform to two-electron (alkali-earth and alkali-earth-like) atoms such as Sr and Yb. Alkali atoms remain the foundational choice because of their strong cycling transitions, mature laser-cooling and state-preparation/manipulation methods, and convenient hyperfine qubits. Two-electron atoms offer complementary capabilities, including narrow intercombination and optical-clock transitions, nonmagnetic ground states in bosonic isotopes, and, in the case of Yb, telecom-band transitions for fiber-integrated atomic-qubit interfaces [121]. These features are attractive for long-lived optical memories, precision spectroscopy, and fiber-network-compatible quantum links, but they also impose stringent engineering requirements, including suppression of differential AC-Stark and surface-induced shifts. As a key demonstration, Kestler et al. [200] realized state-insensitive trapping of $^{88}$Sr atoms around an ONF for the 7.6-kHz-wide $5s^2\,{}^1S_0-5s5p\,{}^3P_1$ intercombination transition, using a two-color double-magic-wavelength trap at an atom-surface distance of about 300 nm. This result marks an important step toward ONF interfaces based on alkaline-earth optical-clock transitions.

The narrow intercombination and clock transitions can be utilized for precision metrology. Although quantum-enhanced metrology with ONF platforms remains less developed than their quantum-network applications, efficient guided-mode coupling enables minimally destructive collective readout, a capability that could be used for measurement-induced spin squeezing, enhanced Ramsey spectroscopy, or matter-wave inertial sensors.

### Nanofiber waveguide QED

Nanofiber-based waveguide QED has emerged as a versatile platform for achieving highly efficient light–matter coupling without the requirement of a high-finesse optical cavity. The foundation of nanofiber waveguide QED was established through the implementation of two-color evanescent field traps [59]. This technique allows for the stable trapping of large atomic ensembles—reaching up to several thousand atoms—within the periodic potential minima (lattice sites) formed near the nanofiber surface. In these systems, the periodicity of the atomic array is intrinsically defined by the wavelengths of the trapping lasers, which serves as a decisive factor in determining the phase-matching and interference conditions for the guided modes.

Two experiments demonstrated the formation of atomic mirrors and showed coherent backscattering from ONF-trapped atoms. Sørensen et al. [201] imprinted the required periodicity onto *∼ *1000 trapped Cs atoms via optical pumping with a resonant standing wave, observing *∼ *12% guided-mode reflectivity, two orders of magnitude above that from randomly positioned atoms. Corzo et al. [202] instead used near-resonance trapping beams whose period is nearly commensurate with the resonant wavelength, and with *∼ *2000 Cs atoms achieved reflectances of up to 75%, demonstrating that an ordered atomic chain can function as a high-efficiency fiber-integrated Bragg mirror.

Experiments have demonstrated the relevance of ONFs for use in an all-fiber quantum network. Two back-to-back independent experiments [203, 204] demonstrated EIT-based storage and on-demand retrieval of fiber-guided single-photon level pulses. Sayrin et al. [203] achieved a storage time of $2~{\mu }$s with an efficiency of *3%* in using a trapped array of Cs atoms. Gouraud et al. [204] showed a storage efficiency of 10% in an ensemble of untrapped cold Cs cloud surrounding the ONF. Together, these experiments established that ONF-coupled atomic ensembles can function as fiber-integrated quantum memories.

Solano et al. [205] demonstrated collective emission of Rb atoms coupled to a nanofiber. They observed a super-radiant burst followed by a sub-radiant tail for an ensemble of a few atoms separated by hundreds of micrometers. The key point is that the guided mode mediates an effective dipole–dipole interaction with a range set by the ONF rather than the wavelength, enabling collective effects from macroscopically separated atoms.

Prasad et al. [206] demonstrated that even a weakly coupled ensemble of atoms on a nanofiber can produce strongly correlated photons through collective nonlinear enhancement. They showed that by adjusting the atom number, they could tune the photon statistics from anti-bunching to bunching of up to $g^{(2)}(0)=22$. Hinney et al. [207] further observed quadrature squeezing exhibited by the transmitted light in that system.

Pennetta et al. [208] studied collective radiative effects in a cold Cs atoms coupled to a nanofiber. Using time-resolved transmission and reflection measurements of a single optical pulse propagating through the atoms, they traced the build-up of collective atom–photon correlations. Pennetta et al. [209] also observed coherent coupling between super- and sub-radiant states of a trapped array of Cs atoms collectively coupled to a nanofiber. They showed that an ensemble prepared in a timed Dicke state evolves through sub-radiant states, resulting in an aperiodic drop in the collectively emitted power. This work provides a fundamental insight into collective atom–light coupling.

Another avenue of exploration is exploiting the chiral nature of the atom–light coupling in ONF systems, which has applications in quantum information transfer channels and non-reciprocal optical devices. Recall the inherent local elliptic polarization in the evanescent field, which is essentially the spin-momentum locking of the guided field i.e. the handedness of the ellipticity is inherently locked to the propagation direction and reverses with it. An atom therefore couples differently to the right- and left-propagating guided modes depending on its internal quantum state. Petersen et al. [210] used the spin-orbit coupling of light in an ONF to control the directionality of the scattering process using a gold nanoparticle as a scatterer. Mitsch et al. [211] demonstrated quantum state-controlled directional emission with ONF-trapped Cs atoms. By optically pumping into specific Zeeman sublevels and exciting them with a circularly polarized free-space beam, they controlled the directionality with *∼ * 90% efficiency. In a similar setup and by initializing the trapped atoms into a specific Zeeman state, Sayrin et al. [212] demonstrated direction-dependent high isolation in transmission of the guided light. This is analogous to an optical isolator controlled by the initialization state of the atom. Pucher et al. [213] used a similar configuration to show non-reciprocal Raman amplification of fiber-guided light.

In parallel with studies based on two-color traps, there has been increasing interest in developing techniques that offer higher degrees of control in atomic positioning, independent of the trapping wavelengths. Recent progress with the realization of sub-*λ /2* atomic spacing [66] is one such example. As a promising approach for improved controllability, the integration of optical tweezers has been explored. While the precise control of single atoms using tweezers was initially demonstrated in cavity QED systems [74], recent efforts have extended this capability to multi-atom configurations in waveguide systems. For instance, Takahata et al. have recently reported the use of optical tweezer arrays to position multiple atoms at arbitrary spots near a nanofiber with sub-wavelength precision [62]. These reconfigurable arrays allow for the design of atomic geometries that are decoupled from the guided-mode periodicity of the fiber. Such spatial controllability is currently being considered as a key enabling technology for implementing many-body physics simulations with complex interactions and for developing scalable quantum networks.

The flexibility of the nanofiber platform also facilitates the use of various solid-state emitters as an alternative to cold atoms. The efficient coupling of nanofibers with single quantum dots [214] and the direct placement of two-dimensional materials, such as transition metal dichalcogenides (TMDCs), onto the fiber surface [215] have expanded the scope of waveguide QED. These systems combine the practicality of solid-state devices with the high coupling efficiencies characteristic of nanofiber interfaces.

### Fiber ring resonators containing a nanofiber section

Besides the linear Fabry–Pérot geometry, all-fiber cavities containing a nanofiber section for use in cavity QED experiments have also been demonstrated in the ring geometry [216, 217]. Here, the fibers on each side of a tapered optical fiber section are linked together with a fiber beamsplitter to form a fiber ring resonator. Light can then be coupled into and out of the resonator through the fiber beamsplitter, and atoms can interact with the cavity mode through the evanescent field of the nanofiber section. While the length of these resonators is typically longer than that of fiber Fabry–Pérot resonators, it is possible to maintain large cooperativities even for large resonator lengths, as cooperativity is independent of length [216]. This geometry can therefore present a unique system for studying chiral light–matter interactions [216, 218] as well as the superstrong coupling regime of cavity QED [219–221].

In a proof-of-concept experiment, Jones et al. created a *∼ 2.8* m long ring resonator that could be adjusted to be in a single pass configuration by disconnecting fiber connectors within the resonator [222]. The resulting resonator had an FSR of *∼ 76* MHz and a finesse of $\mathcal {F}\sim 3.6.$ They showed that hot rubidium vapor coupled to the nanofiber section could serve as a loss mechanism, and investigated the nonlinear response at nanowatt power levels.

Schneeweiss et al. demonstrated a 2.35 m long fiber ring resonater with a nanofiber section by incorporating a variable fiber beamsplitter, and achieved a finesse of $\mathcal {F}=75\pm 1$ with an FSR of 87.5 MHz [216]. They predicted that a single cesium atom trapped approximately 200 nm from the nanofiber surface could achieve a cooperativity of *C≈ 1*, and hence that these cavities could enter the strong coupling regime. They also predicted that for cavity lengths exceeding 4 m, the collective coupling strength of atoms coupled to the nanofiber section would exceed the free-spectral range, and the system could enter the regime of superstrong coupling [219], potentially enabling atom-mediated interactions between differing optical modes.

**Fig. 11:**
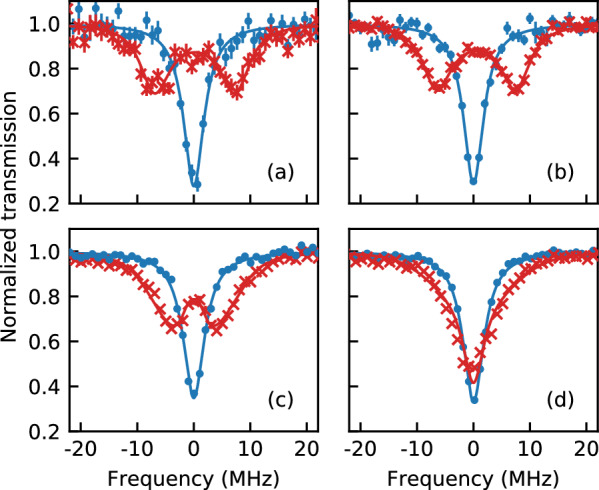
Collective strong coupling of cesium atoms to a fiber ring resonator containing a nanofiber section. Normalized transmission for various input probe powers $P_\textrm{in}$, for the case of atoms interacting with the cavity mode (red crosses), and for an empty cavity (blue circles). **a** $P_\textrm{in} = 30$ pW, **b** $P_\textrm{in} = 60$ pW, **c** $P_\textrm{in} = 750$ pW, and **d** $P_\textrm{in} = 2.3$ nW. Reprinted figure with permission from [217]. *\copyright * Optica Publishing Group

Ruddell et al. demonstrated collective strong coupling of cesium atoms to a nanofiber-section fiber ring resonator [217]. The resonator length was 1.4 m, corresponding to an FSR of 148 MHz, and had a resulting finesse of $\mathcal {F}\approx 35$. In order to stabilize the cavity resonance frequency to the atomic transition, they coupled *∼ 2* mW of far-detuned light at 780 nm into the resonator and utilized the thermal properties of the nanofiber to precisely control and stabilize the resonator length. By overlapping the nanofiber section with a cloud of cold cesium atoms and probing the atomic transition at low power, they were able to observe Rabi-splitting of the cavity resonance, as shown in Fig. 11. As the probe power was increased, the atoms were saturated and the lineshape of the cavity resonance approached that of the empty cavity. Due to having a single-atom cooperativity *C<1*, the splitting was attributed to a collective enhancement with multiple atoms interacting with the cavity mode. Here, the calculated intra-cavity saturation photon number of *∼ 13* photons corresponded to an effective atom coupling rate of 0.6 MHz, with an overall collective cooperativity of 1.5, for an estimated 64 atoms coupled to the cavity mode.

Johnson et al. constructed a *∼ 30* m long fiber ring resonator containing a nanofiber section, and were able to observe superstrong coupling—where the atom–field coupling strength is comparable to or exceeds the FSR—of cesium atoms with as few as 200 atoms [220]. The FSR of their resonator was 7.1 MHz, with an intrinsic single-atom cooperativity of 0.13. By increasing the number of cesium atoms up to *645± 109* atoms, they were able to observe a collectively enhanced atom–field coupling strength of 1.3 times the FSR. In the context of this experiment, Blaha et al. theoretically explored the validity of the Tavis–Cummings model, and showed apparent deviations from the experiment. They derived a more general Hamiltonian description, taking into account cascaded interactions of photons with consecutive emitters, and showed that their model could more correctly describe the superstrong coupling of atoms to the resonator [221].

### Nanofiber cavity QED experiments with evanescent traps

#### Strong coupling in an all-fiber nanofiber cavity

A major step toward scalable cavity QED systems compatible with fiber-based quantum networks was achieved by Kato et al. [47], who demonstrated strong coupling between a single trapped atom and a nanofiber cavity. In this work, a nanofiber segment was integrated between two FBG mirrors to form an all-fiber cavity of length $L_{\textrm{cav}} \approx 33~\textrm{cm}$ with a relatively low finesse ($\mathcal {F} \approx 40$), leveraging the strong transverse confinement and large evanescent field of the guided mode. A key feature of this system is that the atom–photon interaction is enhanced by the tight confinement of the nanofiber-guided mode, allowing the realization of strong coupling even for a long cavity with modest finesse.

**Fig. 12:**
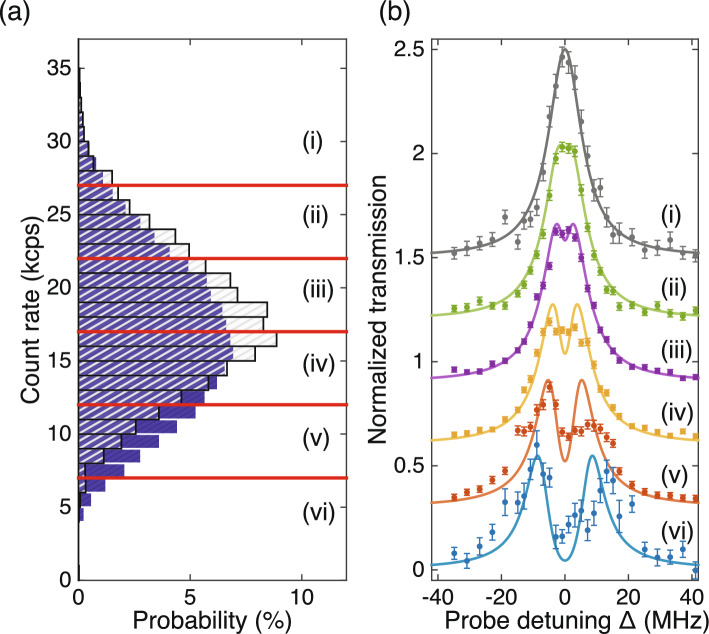
**a** Histogram of the transmission intensity of the detection probe with (blue) and without (gray) the optical molasses. **b** Transmission spectra as functions of the probe detuning *Δ *. Reprinted from Ref. [47]. ©American Physical Society

Cesium atoms were trapped in the evanescent field of the nanofiber using a two-color state-insensitive optical dipole trap, formed by counter-propagating red-detuned beams and a blue-detuned beam at magic wavelengths. The trapping minimum was located approximately $170~\textrm{nm}$ from the fiber surface, ensuring strong overlap between the atomic wavefunction and the guided optical mode. The presence of a single atom coupled to the cavity was detected via a reduction in the transmission of a resonant probe, followed by measurements of the transmission spectra. Clear vacuum Rabi splitting was observed (see Fig. 12), providing direct evidence of coherent atom–cavity interaction. The maximum coupling rate was determined to be $g_0 = 2\pi \times (7.8 \pm 1.2)~\textrm{MHz}$, in good agreement with theoretical estimates based on the cavity mode volume. This exceeds both the cavity field decay rate $\kappa = 2\pi \times 6.4~\textrm{MHz}$ and the atomic polarization decay rate $\gamma = 2\pi \times 2.6~\textrm{MHz}$, thereby establishing the strong coupling regime.

Importantly, it was confirmed that the observed normal-mode splitting originates from a single atom by varying the loading time and showing that the extracted coupling strength remains unchanged while the loading probability decreases. The measured trap lifetime was $\sim 11~\textrm{ms}$, limited by technical heating mechanisms, but sufficiently long to perform spectroscopic measurements. This work represents the first realization of strong coupling in a fully fiber-integrated cavity QED system with a trapped single atom. In contrast to free-space Fabry–Pérot cavities, the all-fiber architecture offers intrinsic compatibility with optical fiber networks, providing a promising route toward scalable quantum networks.

#### Coupled-cavity QED with delocalized photonic modes

An important extension of the above nanofiber-based cavity QED toward distributed quantum systems was demonstrated by Kato et al. [150], who realized a coupled-cavity QED system using two distant nanofiber cavities connected by an optical fiber. In this architecture, each cavity contains an ensemble of atoms coupled to the evanescent field of a nanofiber, while the cavities themselves are coupled via a meter-scale fiber link with losses as low as a few percent. The coherent coupling between the two cavities gives rise to the normal modes delocalized over the entire system. In particular, a central role is played by the so-called *fiber-dark mode*, which is a superposition of the two cavity modes without any excitation in the connecting fiber. This mode enables coherent interaction between distant atoms mediated by delocalized photons.

**Fig. 13:**
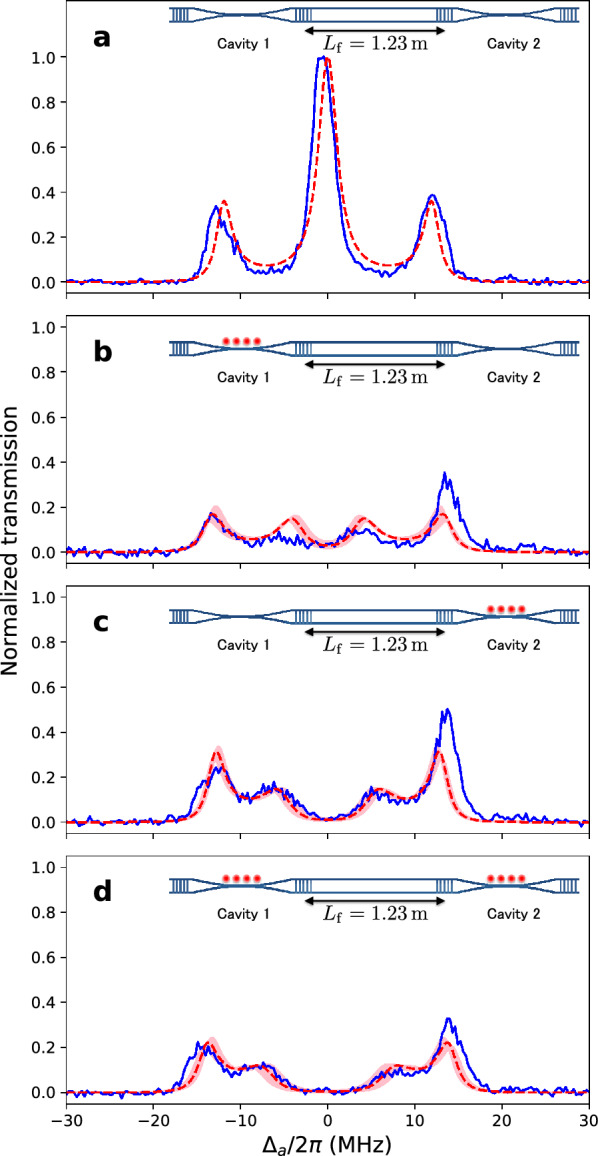
Transmission spectra with different atom-loading conditions. **a**, No atoms are loaded. **b**, Atoms are loaded in cavity 1 only. **c**, Atoms are loaded in cavity 2 only. **d**, Atoms are loaded both cavities. Blue solid lines represent experimental data, while red dashed lines and pink shaded bands are theoretical curves for $(g_{1, \textrm{eff}}, g_{2, \textrm{eff}}) = 2\pi \times (7.2, 7.3)$ MHz and $(g_{1, \textrm{eff}}, g_{2, \textrm{eff}}) = 2\pi \times (7.2 \pm 1.0, 7.3 \pm 1.0)$ MHz, respectively. Reprinted from Ref. [150], licensed under CC BY 4.0

The authors observed the normal-mode structure of the coupled system via transmission spectroscopy. In the absence of atoms, the spectrum exhibits a characteristic triplet structure corresponding to the three photonic normal modes (Fig. 13a). When atoms are loaded into one or both cavities, the central peak associated with the fiber-dark mode undergoes vacuum Rabi splitting (Fig. 13b–d), demonstrating coherent coupling between atoms and the delocalized photonic mode. Notably, when atoms are present in both cavities, the splitting reflects the collective coupling strength $\sqrt{g_{d1}^2 + g_{d2}^2}$, providing clear evidence of dressed states formed by distant atoms interacting through a shared photonic mode.

A key feature of this system is that the effective atom–photon coupling to the fiber-dark mode is independent of the length of the connecting fiber, as confirmed experimentally by varying the fiber length. This highlights the robustness of the delocalized mode against spatial separation and underscores its suitability for quantum networking applications. Furthermore, the system exhibits strong optical nonlinearity at the few-photon level, with saturation photon numbers on the order of unity, indicating its potential for photon-mediated interactions in distributed quantum systems.

This work establishes a scalable platform for realizing coherent interactions between distant quantum nodes in an all-fiber architecture. It provides a direct physical implementation of coupled-cavity QED models and opens new avenues for exploring many-body physics of light and matter, as well as deterministic quantum state transfer and entanglement generation across macroscopic distances.

#### Cavity-dark modes and nonlocal atom–photon interactions

Based on the above realization of coupled-cavity QED systems, White et al. [151] further explored the full normal-modes of such systems and demonstrated the existence of a qualitatively distinct type of dark mode, the *cavity-dark mode*. While previous work focused on fiber-dark modes, which suppress excitation in the connecting fiber, this study identified a complementary mode in which photonic excitation is absent in the two cavities that host the atoms. In this cavity-dark mode, the atoms are instead dressed by photons residing in the remote link cavity, leading to a highly nonlocal form of light–matter interaction.

**Fig. 14:**
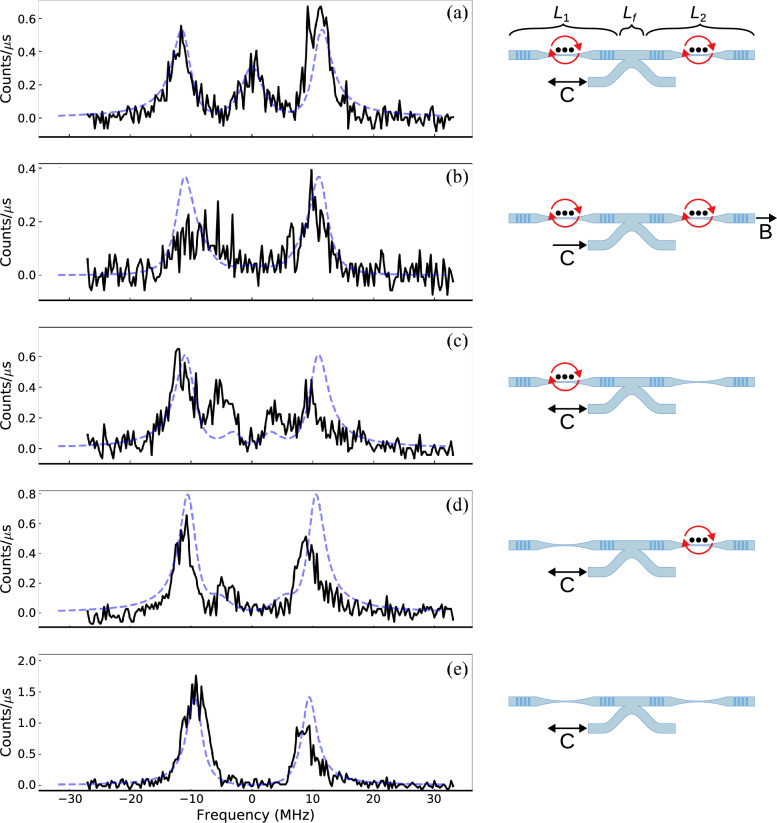
Probing the cavity dark mode. (a)-(d) show data for the spectroscopy driving and detecting at port *C* for ($L_1$, $L_f$, $L_2$) = (0.92, 1.80, 1.38) m and FBG reflectances (0.80,0.65,0.80,0.85). Dashed lines show theoretical calculations. **a** Atoms are in both cavities (*C→ C* spectroscopy). The cavity dark mode is visible as the central 0 MHz resonance. The two symmetric bright modes are also observed. **b** Atoms are in both cavities (*C→ B* spectroscopy). Only the two bright modes are observed. **c** Atoms are in Cavity 1 only (*C→ C* spectroscopy). Four normal modes are observed. **d** Atoms are in Cavity 2 only (*C→ C* spectroscopy), and four normal modes are observed. **e** Empty cavity spectra (*C→ C* spectroscopy), where two normal modes are observed. Corresponding schematics detailing each experimental condition are shown on the right. Adapted from Ref. [151]. © American Physical Society

Access to this mode was enabled by introducing a fiber beam splitter into the linking fiber as a tapping port, allowing direct excitation and detection of the link-cavity field. Spectroscopic measurements (Fig. 14) revealed a clear resonance at zero detuning corresponding to the cavity-dark mode, which is absent when probing from the cavity ports. Simultaneous detection at different ports confirmed that this mode does not populate the local cavity fields, consistent with its dark-state character.

A significant consequence of this mode is the realization of remote excitation and nonlocal saturation of atoms. Even though no photons are present at the atomic positions, the atoms are excited via the delocalized photonic field in the link cavity. As the drive power is increased, the system exhibits saturation behavior, leading to a suppression of the on-resonant transmission. This counterintuitive effect highlights the nonlinear response of the system mediated by nonlocal normal modes.

The observation of cavity-dark modes completes the characterization of the normal-mode structure of coupled-cavity QED systems and demonstrates the rich variety of interference effects available in such systems. These results provide deeper insight into the control of light–matter interactions in distributed architectures and point toward novel schemes for quantum information processing, where interactions between distant nodes can be engineered through tailored photonic modes without local excitation.

#### Dynamically controlled photon transport in coupled-cavity QED systems

A further development of nanofiber-based coupled-cavity QED systems is the introduction of dynamic control of photon transport, as demonstrated by Kato et al. [152]. In this work, the authors investigate photon transport through fiber-based cavity arrays in the presence of fast temporal modulation of the atom–cavity interaction, implemented via a time-dependent AC Stark shift of the atomic resonance.

**Fig. 15:**
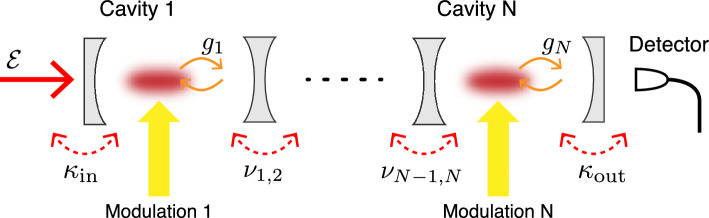
Schematic diagram of the coupled-cavities system. Each cavity contains an atomic ensemble (red ovals), which couples to the cavity mode at the rate of $g_i$. In order to introduce the modulation, an external laser field is applied to induce an AC Stark shift (yellow arrows). Reprinted with permission from Ref. [152] © Optica Publishing Group

The system is described by a driven-dissipative model of coupled cavities interacting with atoms, where the atomic transition frequency is modulated in time with a bandwidth exceeding all intrinsic system rates, including the atom–cavity coupling *g*, the cavity decay rate *κ, *and the inter-cavity coupling $\nu _{ij}$ (see Fig. 15). This fast modulation enables control over the dynamical evolution of photonic excitations and, consequently, over the transmission properties of the system.

In the single-cavity case, the modulation induces sidebands in the dressed-state spectrum of the atom–cavity system, allowing resonant enhancement of transmission when the modulation frequency compensates the detuning between the probe field and the dressed states. The experimentally observed transmission enhancement peaks occur at modulation frequencies corresponding to the atom–cavity coupling strength, in agreement with numerical simulations based on master equations.

Extending to coupled-cavity configurations, the authors show that the transport properties are sensitive to the coupling regime between cavities. In the weak-coupling regime ($\nu _{12} \ll \kappa$), the system behaves effectively as independent cavities, and the enhancement peak remains determined by the local atom–cavity coupling. In contrast, in the intermediate regime ($\nu _{12} \sim \kappa$), where delocalized normal modes are formed, the enhancement peak shifts to a frequency determined by the hybridized eigenmodes of the coupled system, $\sqrt{g^2 + \nu _{12}^2}$. This shift directly reflects the transition from localized to delocalized photonic states.

These results demonstrate that dynamic modulation provides a powerful tool for controlling photon transport in cavity QED networks. Unlike static disorder or fixed coupling architectures, the use of fast temporal modulation allows real-time tuning of transport properties and access to regimes where coherent interference and nonlinearity interplay.

### Nanofiber cavity QED experiments with side-illumination nano-traps

A single atom deterministically interfaced with a nanofiber cavity offers significant advantages over probabilistic single-atom schemes [55]. A side-illumination trap with a nanofiber is a suitable technique to achieve this. In this context, a significant milestone was achieved by Nayak et al. [74] in which the authors demonstrated a deterministic method for interfacing a single atom with a nanofiber cavity. A single Cs atom was trapped near the nanofiber section of a nanofiber cavity, and its fluorescence was monitored in real time via the guided mode of the fiber. This observation demonstrates the feasibility of nanofiber-based systems as a platform for all-fiber cavity QED.

**Fig. 16:**
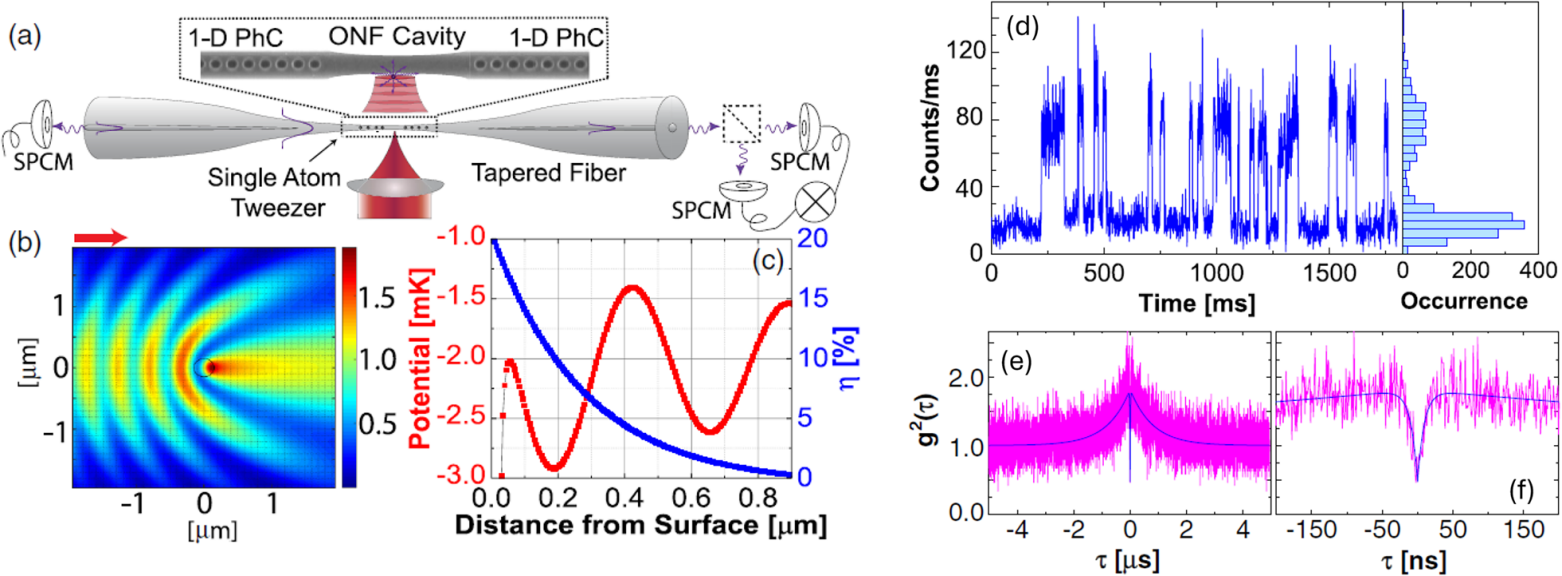
**a** Schematic of the experimental system, PhC: photonic crystal, SPCM: single photon counting module. **b** Intensity distribution around the ONF. **c** The estimated trapping potential and coupling efficiency, *η * (referred as *β * in main text). **d** Typical photon count measured through the ONF (e,f) photon correlation of the fluorescence signal through ONF. Reprinted from Ref. [74] © The American Physical Society

A schematic of this configuration is shown in Fig. 16a. A cavity structure was realized by integrating two PhC mirrors (Sect. 3.3) on either side of the nanofiber section. The nanofiber waist region had a diameter of 300 nm, and the PhC structures were fabricated on the 500 nm diameter nanofiber sections on either side of the trapping region. The cavity formed between these PhC structures exhibited a finesse of 140 (*κ = 164* MHz) and FSR of *23± 2* GHz, corresponding to an effective cavity length of $L_\textrm{eff}= 6.5\pm 0.5$ mm.

A 14 mW red-detuned laser at 938 nm, the magic wavelength for Cs atoms, was used to create a trapping beam with a 1 *μ *m beam waist focused onto the nanofiber, forming a trap with an estimated depth of 0.9 mK approximately 190 nm away from the nanofiber surface. The field pattern and potential experienced by the Cs atom are shown in Fig. 16b and c, respectively. Even though multiple trap sites were formed, only the first trap site sufficiently overlapped with the evanescent field of the guided mode and enabled coupling of the fluorescence into the guided mode, which is evident from the coupling efficiency plotted in Fig. 16c. A step-like behavior in the fluorescence from the trapped atom, detected through the guided mode of the fiber, was observed, with clear steps of *62± 13* counts above the background, as shown in Fig. 16d, indicating the presence of a single atom in the trap. Photon correlation measurements confirmed the single-atom nature, with clear anti-bunching in the fluorescence signal characterized by $g^{2}(0) = 0.47$, as shown in Fig. 16e. The authors also observed bunching behavior along with a central anti-bunching behavior on a larger time scale, as shown in the Fig. 16f. This corresponds to clustering of the photon emission into the cavity mode as observed and explained in Ref. [223]. The lifetime of the atom in this trap was measured to be *52± 5* ms, which is consistent with typically observed values.

A cavity-enhanced coupling efficiency of *85± 2*% was measured while the nanofiber coupling efficiency alone was estimated to be *∼ 10*%, clearly demonstrating the strong effect of the cavity. The atom–cavity coupling rate was estimated by using the dependency of fluorescence photon statistics on the cavity QED parameters of the different cavity modes with varying finesse. The single atom–cavity coupling rate was measured to be $g_0/(2\pi )=34\pm 2$ MHz, which corresponds to a cooperativity of *C=5.4± 0.6* and a cavity enhanced Purcell factor of *P = 6.4 ± 0.6*. The authors attributed the deviation of *g* from the theoretically estimated value of *63± 3* MHz, to the weak confinement of the atom along the fiber axis. The importance of further cooling of the atoms in the trap is evident [224, 225], and the authors suggest that this cavity, with improved finesse, holds strong promise for fiber-integrated quantum photonics.

## Conclusion

Optical nanofibers have emerged as a distinctive platform for cavity and waveguide quantum electrodynamics. Their subwavelength cross section simultaneously provides strong transverse confinement of guided light, a large evanescent field accessible to nearby atoms, and intrinsically efficient coupling to standard single-mode optical fibers. As a result, nanofiber systems unify two complementary routes to strong light–matter interaction: cavity-enhanced interactions in fully fiber-integrated architectures and waveguide-mediated interactions in one-dimensional geometries.

As reviewed in this article, steady progress in fabrication, cavity engineering, and atom trapping has transformed nanofiber systems from sensitive probes of cold atoms into versatile quantum interfaces. Ultra-low-loss tapers, fiber Bragg grating cavities, nanofiber photonic-crystal resonators, and state-insensitive evanescent-field traps have enabled efficient interfacing of guided photons with trapped atoms. These advances have led to the observation of strong coupling in all-fiber cavities, collective and chiral effects in nanofiber waveguide QED, coherent interactions between distant atoms mediated by coupled cavities, dynamically controlled photon transport, and deterministic single-atom trapping using side-illumination schemes. Taken together, these results establish optical nanofibers as a realistic platform for quantum nonlinear optics, distributed quantum systems, and fiber-integrated quantum interfaces.

At the same time, several challenges remain before nanofiber cavity and waveguide QED can fully realize its potential in large-scale quantum technologies. Important subjects include further extension of trap lifetimes and coherence times, improved motional control and near-ground-state cooling, deterministic preparation of single atoms and engineered atom arrays, and stable simultaneous control of multiple coupled cavities. In parallel, the integration of reconfigurable optical tweezers and nanofibers will strongly improve controllability and scalability of this platform.

For future prospects, optical nanofibers are expected to serve as a bridge between fundamental quantum optics and emerging quantum technologies. Their natural compatibility with fiber networks makes them attractive for quantum memories, single-photon sources, photon-mediated quantum gates, and modular quantum-network nodes. At the same time, their one-dimensional guided geometry provides a powerful setting for exploring collective emission, chiral interactions, engineered many-body states, and nonequilibrium photon transport. With continued advances in fabrication, trapping, and coherent control, nanofiber cavity and waveguide QED are likely to play an increasingly important role in the development of scalable quantum photonic platforms.

## Data Availability

Not applicable.

## Abbreviations

AC: Alternating current
CPF: Controlled phase flip
CZ: Controlled-Z
DC: Direct current
DUV: Deep ultraviolet
EIT: Electromagnetically induced transparency
FBG: Fiber Bragg grating
FDTD: Finite-difference time-domain
FIB: Focused ion beam
FSR: Free spectral range
FWHM: Full width at half maximum
HWP: Half-wave plate
LP: Linearly polarized
MOT: Magneto-optical trap
ONF: Optical nanofiber
PBS: Polarizing beam-splitter
PhC: Photonic crystal
QED: Quantum electrodynamics
QWP: Quarter-wave plate
SEM: Scanning electron microscope
TE: Transverse electric
TM: Transverse magnetic
TMDC: Transition metal dichalcogenide
UV: Ultraviolet
vSTIRAP: Vacuum-stimulated Raman adiabatic passage
